# Cross-kingdom RNA interference promotes arbuscular mycorrhiza development

**DOI:** 10.1038/s41477-026-02247-2

**Published:** 2026-03-11

**Authors:** Annika Usländer, Manisha V. Haag, An-Po Cheng, Bernhard Lederer, Jin Yan Khoo, Florian Dunker, Ivan F. Acosta, Arne Weiberg, Caroline Gutjahr

**Affiliations:** 1 Max-Planck-Institute of Molecular Plant Physiology, Potsdam-Golm, Germany; 2 Plant Genetics, TUM School of Life Sciences, Technical University of Munich, Freising, Germany; 3 Faculty of Biology, Genetics, University of Munich, Martinsried, Germany; 4 Institute of Plant Science and Microbiology, Department of Biology, University of Hamburg, Hamburg, Germany

**Keywords:** Arbuscular mycorrhiza, RNAi

## Abstract

Cross-kingdom RNA interference is an emerging concept in plant–pathogen interactions. Here we provide evidence that cross-kingdom RNA interference also occurs in a beneficial plant symbiosis called arbuscular mycorrhiza. The arbuscular mycorrhizal fungus *Rhizophagus irregularis* transfers small RNAs into plant cells, promoting the colonization of host roots. This finding establishes inter-organismal RNA communication as a new regulatory mechanism of this ancient and widespread symbiosis.

## Abstract (web summary)

The authors provide evidence that symbiotic fungi forming widespread arbuscular mycorrhiza symbioses use cross-kingdom RNA interference to silence plant genes and promote their colonization of host roots.

## Main

The roots of about 80% of land plants are colonized by fungi of the Glomeromycotina to form arbuscular mycorrhiza (AM) symbioses, which increase plant nutrition and performance and therefore raise interest for application in sustainable agricultural practices^1,2^. The fungi collect mineral nutrients from the soil, transport them into the roots and release them to plants via highly branched hyphal structures, the arbuscules, which form inside root cortex cells; in turn, they receive organic carbon in the form of sugars and lipids from plant hosts^3^. A plant signalling network encoded by so-called common symbiosis genes ensures a compatible interaction and accommodation of the fungus inside root cells^4^. Still, it appears that AM fungi need to additionally overcome plant defences at the initial stages of colonization and in arbuscule-containing cells, which may be elicited through chitin and/or β-glucan fragments peeling off their cell walls^5–7^.

Cross-kingdom RNA interference (ckRNAi) is an emerging concept in plant–microbe interactions in which fungal and oomycete pathogens deliver small RNAs (sRNAs) into plants to promote infection^8,9^. These pathogen sRNAs load into the plant’s own Argonaute(AGO)/RNA-induced silencing complex to silence host mRNAs with functions in plant defence. ckRNAi is bidirectional, as plants too transfer sRNAs into attacking pathogens for defence^10^. Rhizobial bacteria engaging in root nodule symbiosis and ectomycorrhizal fungi have also been found to release sRNAs to host roots to support colonization and symbiosis^11,12^, and one sRNA from *Rhizophagus irregularis* (Rir*2216*) was observed to transfer into *Medicago truncatula* roots, silencing a WRKY transcription factor to promote symbiosis^13^. It is thus an appealing hypothesis that inter-organismal RNA communication occurred in ancient AM symbiosis, which evolved about 450 million years ago, although AM fungi belong to a different clade (the Glomeromycotina) and have a different lifestyle than pathogenic fungi. Here we probed for evidence of ckRNAi in AM using the model fungus *R. irregularis* during root colonization of the model legume *Lotus japonicus*.

**Fig. 1:**
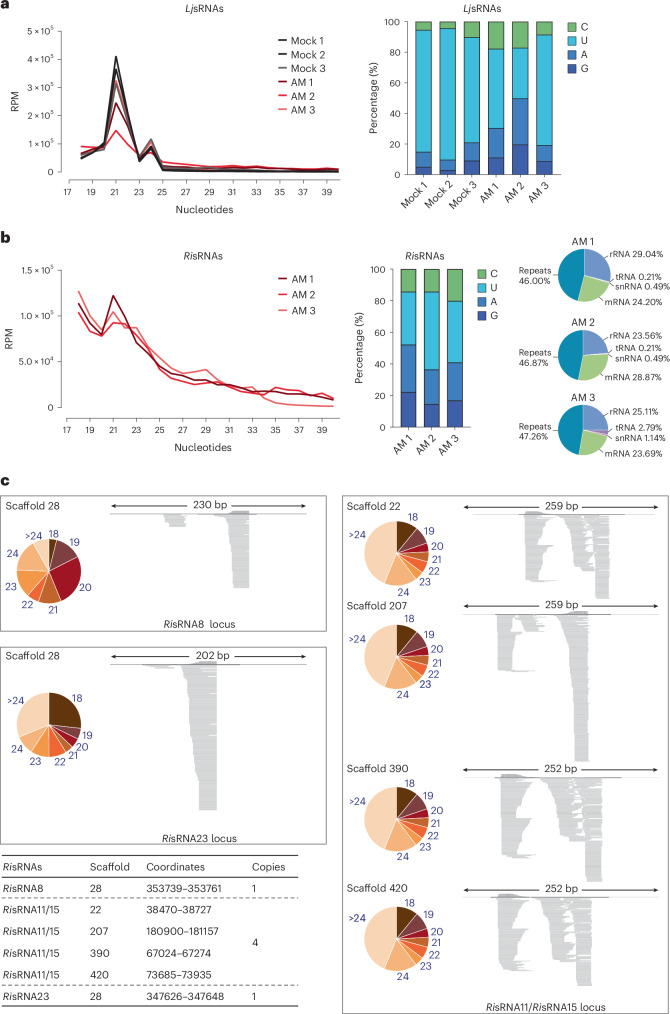
*R. irregularis* sRNAs are associated with *Lj*AGO1. **a**, Size profiles and 5′-terminal nucleotide distributions of *Lj*AGO1-associated *Lj*sRNAs from control (mock) and *R. irregularis*-colonized (AM) roots harvested at seven weeks post inoculation. Three independent experiments (one replicate each) of co-IP sRNA sequencing were conducted. RPM, reads per million. **b**, Size profiles and 5′-terminal nucleotide distributions of *Lj*AGO1-associated *Ri*sRNAs of the samples shown in **a**. The pie charts show relative read counts of *Lj*AGO-associated *Ri*sRNAs mapped to distinct regions of the *R. irregularis* genome. snRNA, small nuclear RNA. **c**, Size distributions of *Ri*sRNA8, *Ri*sRNA11, *Ri*sRNA15 and *Ri*sRNA23 and *R. irregularis* genome coordinates to which these sRNAs perfectly align. *Ri*sRNA11 and 15 aligned to four genomic locations. Source data

To identify candidate *R. irregularis* sRNAs that potentially induce ckRNAi in roots, we analysed the sRNA load of *L. japonicus* AGO1 during root colonization via AGO1 co-immunoprecipitation (co-IP). We used the Agrisera anti-AGO1 antibody raised against the amino-terminal part of *Arabidopsis thaliana* AGO1. A phylogenetic tree displayed a close relationship between *At*AGO1 and *Lj*AGO1 and a large distance to AGO proteins from *R. irregularis* (Supplementary Fig. 1 and Supplementary Data 1). To evaluate cross-reactivity with *Lj*AGO and *Ri*AGOs, we performed a multiple sequence alignment of the N-terminal part of AGOs, including *At*AGO1 (At1G48410), *Lj*AGO1 (LotjaGi2g1v0183100), the two *Lotus* AGOs LotjaGi5g1v0309200 and LotjaGi6g1v0002100 that cluster with *At*AGO10, and three *Ri*AGOs (UniProt entries A0A2H5TEZ5, A0A2H5R841 and A0A2H5TEY9). The PIWI domains are highly conserved across AGOs from all three organisms. However, while the N-terminal region of AGO1 is conserved across *A. thaliana* and *L. japonicus*, it is not for *Ri*AGOs, thus allowing for specific pull-down of *Lj*AGO (Supplementary Fig. 2). Nevertheless, to probe for potential cross-reactivity with fungal AGO proteins, we conducted *Lj*AGO-IP from either non-colonized (mock) or *R. irregularis-*colonized (AM) *L. japonicus* roots followed by mass spectrometry (MS)-based proteomics (Supplementary Fig. 3 and Supplementary Data 2). In two co-IP experiments with independently grown plants that were processed on different days, we detected a number of *L. japonicus* and *R. irregularis* proteins, but only one AGO protein, *Lj*AGO1. This confirms that the *Lj*AGO co-IP was successful and the Agrisera anti-AGO1 antibody is specific, at least within the limits of detection by MS. We then successfully performed *Lj*AGO co-IP from non-colonized (mock) or *R. irregularis-*colonized (AM) *L. japonicus* roots for the detection of sRNAs^8,14^, as verified via *Lj*AGO1 immunoblot analysis and by detecting two *L. japonicus* microRNAs, *Lj*miR166 and *Lj*miR396, via stem–loop reverse transcription (RT)-PCR (Supplementary Fig. 4a,b). To generate high-confidence results, we conducted four entirely independent experiments, for which the plants were grown in four different periods, in two different locations (TUM, Freising, experiments 1 and 2; and MPI of Molecular Plant Physiology, Potsdam, experiments 3 and 4) and by two different persons. The AGO1 pulldown, library constructions and sequencing were performed separately and also by two different persons. The fourth experiment was represented by two biological replicates (Supplementary Fig. 4a). Upon cloning and sequencing of sRNAs, we achieved depths of 10–49 million reads per sample using Illumina-based deep sequencing (Supplementary Fig. 5a). We found a size enrichment of 21-nucleotide reads with 5′-terminal uracil (U) preference for sRNAs that mapped to a *L. japonicus* reference genome (Gifu_v1.2) (Fig. 1a and Supplementary Fig. 5b). This was in accordance with sRNA sequencing data obtained from *At*AGO1 co-IP experiments in previous studies^15^. Interestingly, while some variation in the size profile of sRNAs was detected among the four independently grown experiments (Fig. 1a and Supplementary Fig. 5b), the two replicates of experiment 4, which were grown and processed in parallel, showed the exact same size profile, indicating that the common practice of growing and processing samples for omics experiments in parallel leads to less variation (leading to reduced false negatives) but is probably less stringent (leading to more false positives).

We mapped reads that did not perfectly match the *L. japonicus* reference to a reference genome of *R. irregularis* (strain DAOM_181602) and identified 0.09–3.6% of reads per sample that occurred in the *R. irregularis*-colonized but not in the non-colonized root samples and mapped to the fungal genome. *R. irregularis* sRNAs (*Ri*sRNAs) displayed 21–24-nucleotide size enrichment and 5′-terminal U preference (Fig. 1c and Supplementary Fig. 5c). We were thus confident that *Ri*sRNAs loaded into *Lj*AGO during root colonization. We detected another peak of 17-bp sRNAs, of which the relevance remains unknown (Fig. 1c and Supplementary Fig. 5c). When mapping *Ri*sRNAs to distinct annotated *R. irregularis* gene loci, we found that most sequences were derived from DNA repeat regions, including transposons and simple inverted and tandem repeats, while the second most abundant exact hits were mRNAs (pie charts in Fig. 1c and Supplementary Fig. 5c). This result is in accordance with a previous finding that cross-kingdom sRNAs of the fungal pathogen *Botrytis cinerea* were mainly retrotransposon-encoded and 21–22 nucleotides in size and had 5′-terminal U preference^9,15,16^. Considering *Ri*sRNAs in the size range of 21–24 nucleotides with >25 reads per million in all five *Rhizophagus*-colonized *Lj*AGO co-IP samples, using stringent criteria (a free energy ratio cut-off of 0.7 and a score cut-off of 4.5), we predicted 23 *L. japonicus* target genes for 12 *Ri*sRNAs that were associated with *Lj*AGO1 in all five samples (Supplementary Fig. 6a and Supplementary Data 3 and 4). These targets included candidates potentially involved in plant defence (Supplementary Fig. 6a and Supplementary Data 4). Of the 12 *Ri*sRNAs, we chose *Ri*sRNA8, *Ri*sRNA11, *Ri*sRNA15 and *Ri*sRNA23 for further validation, as they were predicted to target mRNAs potentially involved in defence, including an *RVP1-like Toll interleukin receptor-nucleotide binding-leucine rich repeat* disease resistance gene (*TIR-NB-LRR*, *LotjaGi3g1v0422400*), a *Pectin esterase* (*LotjaGi1g1v0285700*), a *Flavin-containing monooxygenase* (*LotjaGi1g1v0192600*), a *Cyclic Nucleotide Gated Channel* (*CNGC*, *LotjaGi1g1v0792000*) and a *NTM1-like 9 NAC-domain transcription factor* (*LotjaGi1g1v0301000*). *Ri*sRNA8 and *Ri*sRNA23 were encoded by a single intergenic locus each, and *Ri*sRNA11 and *Ri*sRNA15, which differ only by a single nucleotide, were encoded by repetitive elements at four genomic loci. These loci indicated some peaks for sRNA production of various sizes (Fig. 1c). We performed expression analysis of predicted target genes of *Ri*sRNA8, *Ri*sRNA11, *Ri*sRNA15 and *Ri*sRNA23 using quantitative RT-PCR (RT-qPCR). Several of these target genes showed lower mRNA levels in *R. irregularis*-colonized (65% root length colonization) than in non-colonized *L. japonicus* roots (Supplementary Fig. 6b), supporting the idea that they are downregulated by *Ri*sRNAs. To confirm that *Ri*sRNAs can direct slicing of *L. japonicus* target mRNAs, we performed an in vitro cleavage assay with recombinant *Lj*AGO1 or GFP as a control pre-incubated with either *Ri*sRNA8 or *Ri*sRNA23. Three of the four predicted target mRNAs that were tested as examples were cleaved in the presence of the cognate *Ri*sRNA (Supplementary Fig. 7). Among them was *LotjaGi4g1v0232500*, which showed higher expression in AM roots than in non-colonized roots (Supplementary Fig. 6b), raising the possibility that the accumulation of this mRNA is dampened and may be induced by AM to even higher levels in the absence of *Ri*sRNAs (see also Supplementary Fig 8b). The alignment of *Ri*sRNA23 with the fourth mRNA (*LotjaGi1g1v0792000*) displays a wobble at position 10–11 (Supplementary Fig. 6a), potentially explaining the absence of cleavage in vitro.

**Fig. 2:**
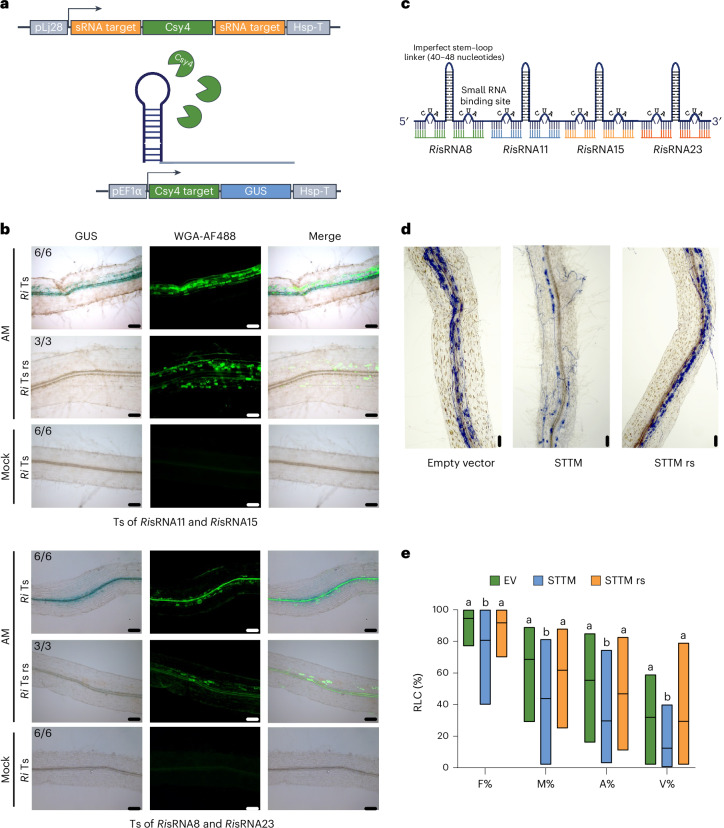
*Ri*sRNAs are transferred to the host plant and promote colonization. **a**, The switch-on ckRNAi reporter construct for detecting *Ri*sRNA-directed gene silencing in *L. japonicus* roots. **b**, Images of *L. japonicus* hairy roots expressing the switch-on ckRNAi reporter upon inoculation with the fungus *R. irregularis* (AM) or without the fungus (mock) at seven weeks post inoculation. Bright-field images of roots expressing the target gene sequences of candidate *Ri*sRNAs (*Ri* Ts) show GUS staining at fungal colonization sites indicated by green-fluorescent WGA-AlexaFluor488 (WGA-AF488) staining. No GUS activation was obtained in roots expressing random scrambled target sequences of candidate *Ri*sRNAs (*Ri* Ts rs) or in roots grown without the fungus. The three image rows at the top display roots expressing the target sequence of the gene *LotjaGi3g1v0422400* targeted by *Ri*sRNA11 and of the gene *LotjaGi1g1v0285700* targeted by *Ri*sRNA15. The images below show roots expressing the target sequence of the gene *LotjaGi1g1v0192600* targeted by *Ri*sRNA8 and of the gene *LotjaGi1g1v0301000* targeted by *Ri*sRNA23. The numbers in the images indicate the numbers of root systems showing the displayed GUS activity per total root systems inspected. The results are from two independent experiments with three root systems each (except the random scrambled control, which was included in the second experiment). Scale bars, 100 µm. **c**, A quadruple STTM construct was designed to simultaneously scavenge the four *Ri*sRNAs *Ri*sRNA8, *Ri*sRNA11, *Ri*sRNA15 and *Ri*sRNA23 in *L. japonicus* hairy roots. **d**, Representative images of *L. japonicus* hairy roots colonized with *R. irregularis* 30 days post inoculation transformed with an empty vector, the quadruple STTM construct (STTM) or the STTM construct with random scrambled sRNA sequences (STTM rs). The fungus was stained with acid ink. Scale bars, 100 µm. **e**, Root length colonization (RLC%) of *L. japonicus* roots with *R. irregularis* was quantified using the Trouvelot method (F%, frequency of colonization; M%, intensity of colonization; A%, arbuscule abundance; V%, vesicle abundance) at five weeks post inoculation after acid ink staining. The bars indicate the range (minimum to maximum) of the biological replicates, with horizontal black lines showing the mean. The experiment was repeated four times independently, and the results were combined. Different letters indicate statistically different groups (ordinary one-way analysis of variance, post hoc Tukey’s test; Replicate 1: *n*_EV_ = 6, *n*_STTM_ = 12, *n*_STTM rs_ = 12; Replicate 2: *n*_EV_ = 8, *n*_STTM_ = 10, *n*_STTM rs_ = 9; Replicate 3: *n*_EV_ = 13, *n*_STTM_ = 12, *n*_STTM rs_ = 13; Replicate 4: *n*_EV_ = 9, *n*_STTM_ = 10, *n*_STTM rs_ = 10; *P* ≤ 0.05; exact *P* values for all comparisons are provided in source data 1). Source data

To directly support ckRNAi during AM development, we designed switch-on ckRNAi reporter constructs and transiently expressed them in *L. japonicus* hairy roots, following the strategy previously applied in plant–oomycete and plant–fungus interactions^8,15^. Briefly, this two-component reporter construct encoded the CRISPR-type RNA nuclease Csy4, which acted as a constitutive repressor of a *GUS* reporter gene. *Csy4* expression was under the control of the promoter of one of the target genes (*LotjaGi1g1v0285700*, annotated as a pectin methylesterase gene), and the GUS reporter was under the control of an *A. thaliana EF1α* promoter. 5′ and 3′ to the *Csy4* transgene, we fused predicted target sequences of two of the candidate *Ri*sRNAs (Fig. 2a). If *Ri*sRNAs were transferred into roots and capable of silencing the predicted *L. japonicus* target sequences, this would lead to the removal of *Csy4* mRNA and activation of the GUS reporter. Hairy roots transformed with the reporter construct were inoculated with *R. irregularis*, and successful colonization was depicted after seven weeks using WGA-AlexaFluor488 staining. For two different combinations of *Ri*sRNA target sites, GUS reporter activity was observed only in colonized tissue—namely, in the vicinity of arbuscules and intraradical hyphae in the inner cortex (Fig. 2b). Non-inoculated roots did not show any GUS activation at any time. To confirm that GUS activation was due to sequence-specific ckRNAi, we expressed a reporter construct that carried scrambled sequences of target sites (Ts resistant, rs) (Supplementary Fig. 6a). Roots expressing Ts rs control constructs did not show any GUS activation despite *R. irregularis* colonization (Fig. 2b). On the basis of this result, we concluded that *R. irregularis* induced ckRNAi in *L. japonicus* roots during AM development.

To understand whether the transferred *Ri*sRNAs are relevant for AM development, we used a short-tandem-target-mimic (STTM) strategy^15,17^ to block *Ri*sRNAs in *L. japonicus* gene silencing. We simultaneously sequestered the four candidate *Ri*sRNAs (*Ri*sRNA8, 11, 15 and 23) by expressing quadruplicates of STTM (Fig. 2c) in *L. japonicus* hairy roots and inoculated these with *R. irregularis*. Compared with empty-vector-transformed control roots, STTM-expressing roots showed reduced levels of *R. irregularis* colonization (Fig. 2d,e and Supplementary Fig. 8a). The effect of *Ri*sRNA sequestration was sequence-specific, because colonization was unaltered in roots expressing an STTM resistant (rs) version carrying scrambled *Ri*sRNA target sites in five independent experiments (Fig. 2d,e and Supplementary Fig. 8a). Sequestration of sRNAs by STTM transcripts should lead to increased expression of putative *Ri*sRNA host target genes, if silencing is achieved via mRNA cleavage. Root colonization by AM fungi is highly dynamic, with constant formation and decay of colonization units that occur synchronously across the root system. Therefore, if AM fungi release sRNAs during specific stages of their intraradical development, this may affect only a small number of cells, thus hampering the detection of changes in target gene expression in mRNA extracted from bulk roots. To examine the expression of putative targets of *Ri*sRNA8, 11, 15 and 23 in the STTM experiment, we considered the dynamic nature of AM development and ran a time course in which we harvested colonized roots at intervals of 2–4 days between 23 and 32 days post inoculation for RNA extraction. Colonization of STTM roots was significantly reduced at most time points (Supplementary Fig. 8a). Expression of the fungal housekeeping gene *RiEF1α* and the plant marker gene for arbuscule-containing cells *LjPT4* was significantly reduced only at the first time point at 27 days post inoculation. A poor correlation of marker gene expression with colonization is common for *L. japonicus* roots at later stages of colonization and probably reflects the relative quantity of colonized cells in which gene expression is ramping up to host the fungus or levelling off during fungal degeneration. It may also be explained by the recently described individuality of arbuscule-containing cells shown in rice in which classical marker genes show alternating expression patterns across different arbuscule-containing cells^18^. Four of the eight tested target genes showed the expected expression pattern, with increased expression in STTM roots at a minimum of one time point (Supplementary Fig. 8b). One of them is a predicted target of *Ri*sRNA8, whereas three are predicted targets of *Ri*sRNA23, suggesting that these two *Ri*sRNAs regulate some of their targets via AGO1-mediated slicing. We detected silencing by *Ri*sRNA11 and/or 15 through the *Csy4* reporter. Whether it was not detectable in the STTM experiment because of their spatio-temporal distribution or because they act by inhibiting translation remains to be determined.

In conclusion, we provide evidence that the AM fungus *R. irregularis* transfers *Ri*sRNAs into root cells of the host plant *L. japonicus* during colonization. These load into *Lj*AGO, thereby inducing ckRNAi. *Ri*sRNAs promote AM symbiosis development, possibly by suppressing mRNAs involved in plant defence and/or other functions with inhibitory effects on root colonization. The four *Ri*sRNAs sequestered by STTM were predicted to target a *TIR-NB-LRR*, a *Pectin esterase*, a *Flavin-containing monooxygenase*, a *CNGC* and a homologue of the *NAC-domain transcription factor* gene *NTM1-like 9*, which could indeed act in blocking root colonization by promoting pattern- and/or effector-triggered immunity^19^, increasing cell wall stiffness^20^, producing toxic or immunity-inducing metabolites^21,22^ or transcriptionally activating genes involved in defence^23,24^. These possibilities raise the question of whether the suppression of plant immunity through ckRNAi could provide an entry gate for pathogens. Our observations with the switch-on RNAi reporter suggest that ckRNAi may occur only in a very localized manner in a limited number of cells close to fungal hyphae and in colonized cortex cells. This would allow plants to maintain defence capacity in most of the outer root cell layers. While the exact location of RNA exchange still needs to be determined, it will also be interesting to uncover how *Ri*sRNAs are transferred into root cells. The pathogenic fungus *B. cinerea* produces extracellular vesicles loaded with sRNAs that can be taken up by *Arabidopsis* cells via clathrin-mediated endocytosis^25^, and highly active vesicle trafficking from plants and fungi has been observed in the peri-arbuscular space, the apoplastic interface between arbuscules and arbuscule-containing host cells^26,27^. It is therefore likely that sRNA exchange also occurs via extracellular vesicles in AM symbiosis. Another interesting question is whether plant extracellular sRNAs also regulate gene expression in AM fungi. Together, answers to these questions help us understand a novel regulatory mechanism of one of the most widespread symbioses on Earth.

## Methods

### Alignment and phylogenetic tree

AGO protein sequences from *A. thaliana* were retrieved from NCBI GenBank. AGO proteins from *L. japonicus* were identified by BLASTp against the LotusBase database *L. japonicus* Gifu v.1.3 Proteins. AGO proteins from *R. irregularis* were identified by BLASTp against the *R. irregularis* DAOM 197198 v.1.0 database Rhiir2_1_GeneCatalog_proteins_20160502.aa (https://mycocosm.jgi.doe.gov/pages/blast-query.jsf?db=Rhiir2_1). Multi-sequence alignment of the full sequences of AGOs was performed using the MUSCLE tool^28^. The full sequence alignment derived was used to construct a phylogenetic tree using MEGA 11 (v.11.0.13) with the maximum likelihood statistical method^29^.

### Plant material, growth conditions and inoculation with AM fungi

The *L. japonicus* ecotype Gifu wild type was used for all experiments. Seeds were scarified with sandpaper and surface-sterilized with sterilization solution containing 10% Klorix and 0.1% SDS. The seeds were germinated on Gamborg B5 (www.duchefa-biochemie.com) at 24 °C for 10 to 14 days. The seedlings were then transferred to pots (six per pot) containing sand and grown at 24 °C constant temperature and 60% air humidity under 16 h light/8 h dark cycles. The plants were watered twice a week with deionized water and fertilized once a week with ½ Hoagland medium as described previously^30^. For AM colonization, plants were inoculated with 500 spores per plant of *R. irregularis* DAOM197198 (Agronutrition).

### AGO1 co-IP, immunoblot analysis and MS

*Lj*AGO1 was co-immunoprecipitated with anti-AGO1 antibody (rabbit polyclonal, Agrisera AS09 527, 1 µg µl^−1^ diluted 1:10,000) using 5 g of *L. japonicus* roots colonized with the fungus *R. irregularis* or without the fungus, as previously described^14^. For immunoblot analysis, 30% of the co-IP fraction was used. Proteins were separated on 8% (w/v) SDS gels and transferred to 0.45-µm Immobilion FL PVDF membranes (Millipore) using the Hoefer Electrophorese-Blotting-System. Membranes were blocked in 5% (v/v) skim milk in 1× PBS and probed with the primary antibody (anti-AGO1 Agrisera, AS09 527; rabbit polyclonal, 1 µg µl^−1^ diluted 1:5,000) in 1% (v/v) milk in PBS-T (PBS, 0.1% Tween-20) overnight at 4 °C. Membranes were washed with PBS-T (PBS, 0.2% Tween-20) and incubated for 1–2 h with the secondary antibody (goat polyclonal anti-rabbit IRdye800, catalogue number 926-32211, 1 µg µl^−1^ diluted 1:5,000, LI-COR) in 1% (v/v) milk in PBS-T (PBS, 0.1% Tween-20) with 0.02% SDS. Bound antibodies were detected by scanning of the membrane using a LI-COR Odyssey Scanning device.

*Lj*AGO1 co-immunocprecipitated proteins were examined via silver staining by using ROTI Black P (Carl ROTH) following the manufacturer’s instructions. MS was performed as previously described^31^. Beads were incubated with 10 ng µl^−1^ trypsin in 1 M urea and 50 mM ammonium bicarbonate for half an hour. Then, the beads were washed with 50 mM ammonium bicarbonate, and the supernatant was digested overnight with 1 mM DTT. Digested peptides were alkylated and desalted prior to liquid chromatography–MS analysis. *Lj*AGO1 co-immunoprecipitated protein mixtures were subjected to nanoflow reversed-phase liquid chromatography (nanoRP-LC)-MS/MS analysis on the Ultimate 3000 nano chromatography system (Thermo Fisher Scientific) equipped with a 25 cm Aurora column (Ionopticks) and coupled to the Orbitrap Exploris-480 mass spectrometer (Thermo Fisher Scientific) operated in data-dependent mode to automatically switch between full-scan MS and MS/MS acquisition. MaxQuant v.2.1.0.0 (ref. ^32^) was used to identify proteins and conduct quantification using iBAQ, and Perseus v.2.0.9.0 (ref. ^33^) was used for display and analysis.

### *Lj*AGO1–sRNA co-IP and sRNA recovery

Small RNAs bound to *Lj*AGO1 were co-immunoprecipitated as described above. The co-immunoprecipitated sRNAs were recovered as previously described^14^. Briefly, 150 μl of sRNA recovery buffer (100 mM Tris-HCl pH 7.5,10 mM EDTA, 300 mM NaCl, 2% SDS, 1 μg μl^−1^ Proteinase K) was added to 450 μl of wash buffer with beads and incubated at 65 °C for 15 min. The mixture was then incubated with 450 μl of phenol for 2 min. Phenol was separated via centrifugation at 13,000 *g*. One phenol/chloroform/isoamylalcohol and two chloroform/isoamylalcohol steps were followed to remove the phenol. Small RNAs were recovered in 0.1× volume 3 M sodium acetate, 2.5× volume 96% ethanol and 20 μg of glycogen (RNA grade) at −20 °C overnight.

### Stem–loop RT-PCR

Small RNAs were detected via stem–loop RT-PCR from 5% of the *Lj*AGO co-IP RNA fraction, as described previously^34^. The primers used in stem–loop RT-PCR are listed in Supplementary Table 1.

### Small RNA cloning, sequencing and target gene prediction

Small RNAs were cloned for Illumina sequencing using the Next Small RNA Prep Kit (New England Biolabs) and sequenced on an Illumina HiSeq1500 platform. The Illumina sequencing data were analysed using the GALAXY Biostar server^35^. Raw reads were de-multiplexed (Illumina Demultiplex, Galaxy v.1.0.0), and adapter sequences were removed (Clip adaptor sequence, Galaxy v.1.0.0). The sequence raw data were deposited at the NCBI SRA server (BioProject ID: PRJNA1099625). The reads were then mapped to the reference genome assemblies of *R. irregularis* (strain DAOM_181602) or *L. japonicus* (Gifu_v1.2, NCBI RefSeq assembly: GCF_012489685.1) using the BOWTIE2 algorithm (Galaxy v.2.3.4.2) in end-to-end alignment mode, without setting the k or a options. Integrative Genomics Viewer was used for displaying the mapping of the target scaffolds^36^. To assign *Ri*sRNA to different types of RNA-encoding genes, the sequence information of *R. irregularis* rRNAs, tRNAs, small nuclear and nucleolar RNAs, and mRNA was downloaded from the Ensembl database. Repeat sequences were used as annotated in a previous study^37^. Reads were counted and normalized on total *R. irregularis* reads per million. Target genes were predicted using the *L. japonicus* CDS inventory (Lotus base, Gifu_v1.3) with the TAPIR tool^38^ using a free energy ratio cut-off of 0.7 and a score cut-off of 4.5.

### Plasmid generation

Promoter regions were amplified using Phusion PCR according to standard protocols with the primers listed in Supplementary Table 1. Plasmids were constructed using Golden Gate cloning^39^ as explained in Supplementary Table 2.

### Hairy root transformation

To generate transgenic hairy roots, *L. japonicus* hypocotyls were transformed with Golden Gate Level III plasmids using transgenic *A. rhizogenes* AR1193 as described previously^40^. Roots were screened for successful transformants using a stereomicroscope (Leica MZ16 FA) using an mCherry fluorescent transformation marker.

### Visualization and quantification of root colonization

*R. irregularis* in colonized *L. japonicus* roots was stained with acid ink^41^ or 0.2 µg ml^−1^ WGA-AlexaFluor488 (Molecular Probes, http://www.lifetechnologies.com/)^42^. Root length colonization was quantified according to Trouvelot et al.^43^ and ×10 magnification using a light microscope (Olympus, CX23). WGA-AlexaFluor488-stained fungal structures were imaged using a fluorescence microscope (Leica DM6 B).

### GUS analysis

*L. japonicus* hairy roots transformed with plasmids containing the *Csy4*-*GUS* reporter constructs were subjected to GUS staining and imaging as previously described^40^.

### Gene expression analysis

For analysis of transcript levels via RT-qPCR, roots were rapidly shock-frozen in liquid nitrogen. RNA was extracted using the Spectrum Plant Total RNA Kit (www.sigmaaldrich.com). The RNA was treated with Invitrogen DNAse I amp. grade (www.invitrogen.com) and tested for purity via PCR. cDNA synthesis was performed with 1 µg RNA using SuperScript IV Reverse Transcriptase (Invitrogen) and the 3′ Oligo-dT primer. Real-time RT-qPCR was performed with qPCR GreenMaster Mix (Jena Bioscience) and with the primers shown in Supplementary Table 1. The qPCR reaction was run on a LightCycler 480 II (Roche). Thermal cycler conditions were 95 °C for 5 min and 45 cycles of 95 °C for 10 s, 60 °C for 10 s and 72 °C for 10 s, followed by dissociation curve analysis. Expression levels were calculated according to the ∆∆Ct method^44^. Each sample was represented by three technical replicates.

### In vitro cleavage assay

RNA in vitro cleavage was performed as previously described^45,46^. Recombinant *Lj*AGO1 and GFP proteins were generated using the *Escherichia coli* Rosetta strain, and proteins were extracted using the Ni Sepharose Excel system (Cytiva). Synthetic phosphorylated *Ri*sRNA was purchased from Merck. mRNA target fragments were produced using the T7 RiboMAXTM Express RNAi System (Promega). 100 nM recombinant *Lj*AGO1 or GFP proteins together with 100 nM *Ri*sRNA and 40 U RiboLock RNase Inhibitor (Thermo Fisher Scientific) were incubated at 26 °C for 90 min. Then, 6 μg of target mRNA and 0.5 μg of yeast RNA were added to the reaction. The reactions were incubated at 26 °C and stopped after 60 min. The products were run on a urea-TAE-acrylamide gel (6% acrylamide, 0.5× TAE, 6 M urea).

### Statistical analysis

Data visualization and statistical analyses were performed in RStudio (www.r-project.org) v.2025.09.2+418 and GraphPad Prism v.10.4.0 (GraphPad Software).

### Reporting summary

Further information on research design is available in the Nature Portfolio Reporting Summary linked to this article.

## Supplementary information

Supplementary Information Supplementary Figs. 1–9.

Reporting Summary

**Supplementary Table 1** Oligonucleotides used in this study, **Supplementary Table 2** Golden Gate plasmids.

Supplementary Data 1 Sequences used for the phylogenetic tree (Supplementary Fig. 1a).

Supplementary Data 2 *L. japonicus* and *R. irregularis* proteins obtained via *Lj*AGO1-IP MS.

Supplementary Data 3 *Lj*AGO-bound *Ri*sRNAs.

Supplementary Data 4 *Ri*sRNAs pulled down in all five AGO1-IPs with predicted *L. japonicus* target genes.

Supplementary Data 5 Number of reads for Supplementary Fig. 5b,c, qPCR data for Supplementary Fig. 6b, per cent root length colonization and qPCR data for Supplementary Fig. 8a,b, and *P* values for all comparisons in Supplementary Fig. 8b.

## Source data

**Source Data Fig. 1** Number of reads, **Source Data Fig. 2** Per cent root length colonization for all parameters and samples.

## Data Availability

The sRNA sequencing raw reads have been deposited in the NCBI Sequence Read Archive database (BioProject ID: PRJNA1099625). Source data are provided with this paper. All other data supporting the findings of this study are available in Supplementary Data 1–4.
