# Supplementary material for: Cross-kingdom RNA interference promotes arbuscular mycorrhiza development
Source: Nat Plants. 2026 Mar 11;12(4):695–702. doi: 10.1038/s41477-026-02247-2 (PMC13106029; doi:10.1038/s41477-026-02247-2)
Supplement: Supplementary file 1 — Supplementary Figs. 1–9. [file 41477_2026_2247_MOESM1_ESM.pdf]

nature plants

Supplementary information https://doi.org/10.1038/s41477-026-02247-2

Cross-kingdom RNA interference promotes arbuscular mycorrhiza development

In the format provided by the authors and unedited

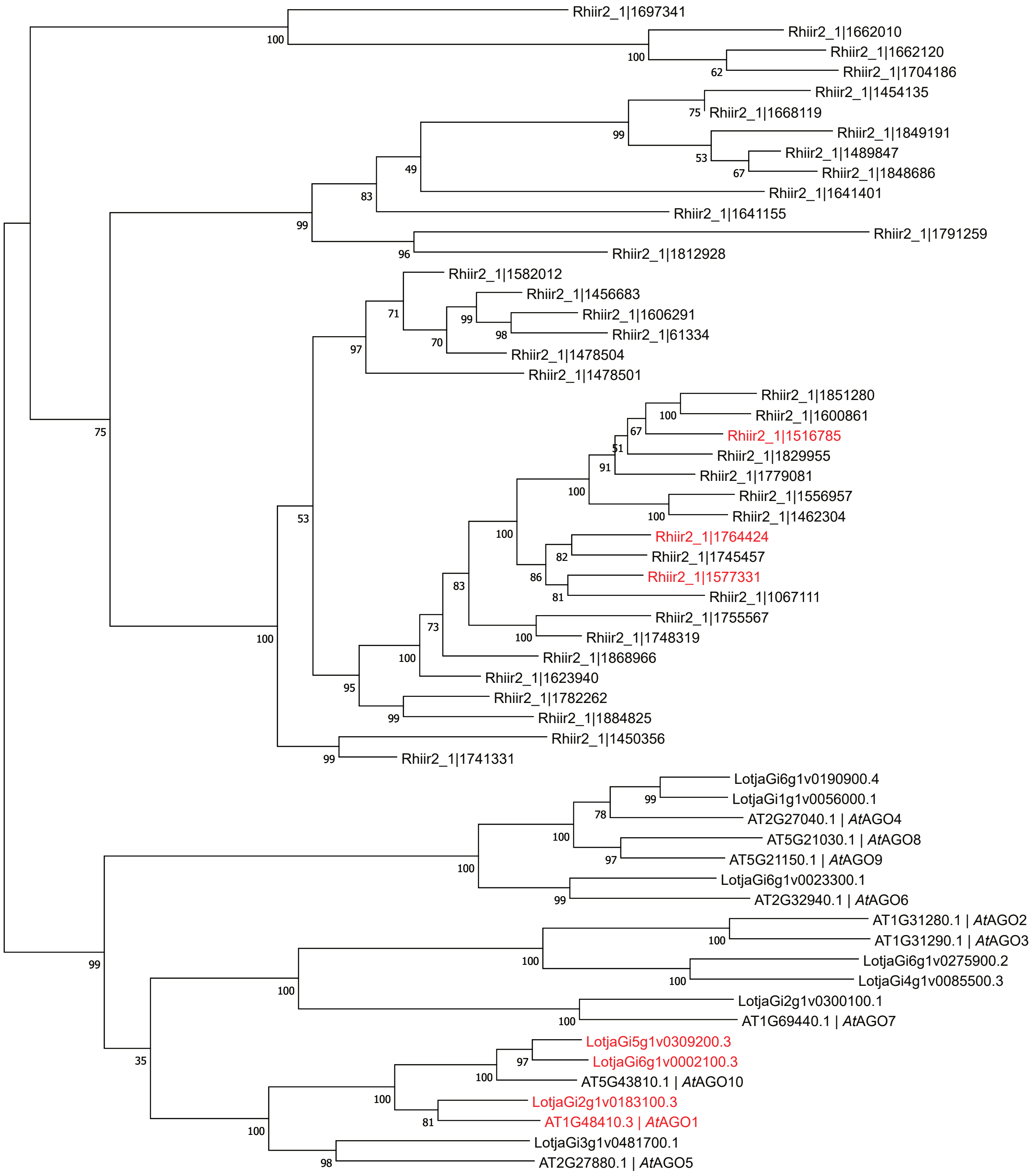

Supplementary Fig. 1| Phylogenetic tree of AGO proteins.
Approximately-maximum likelihood phylogenetic tree of AGO proteins from *L. japonicus*, *A. thaliana* and *R. irregularis*. The tree was constructed using multiple sequence alignment of full-length protein sequences and 500 bootstrap replications. The numbers of *A. thaliana* AGO proteins were adopted from [46] and AGO1 of *L. japonicus* is indicated because it clusters with *A. thaliana* AGO1. Red color indicates the IDs of proteins shown in the alignment in Fig. S2.

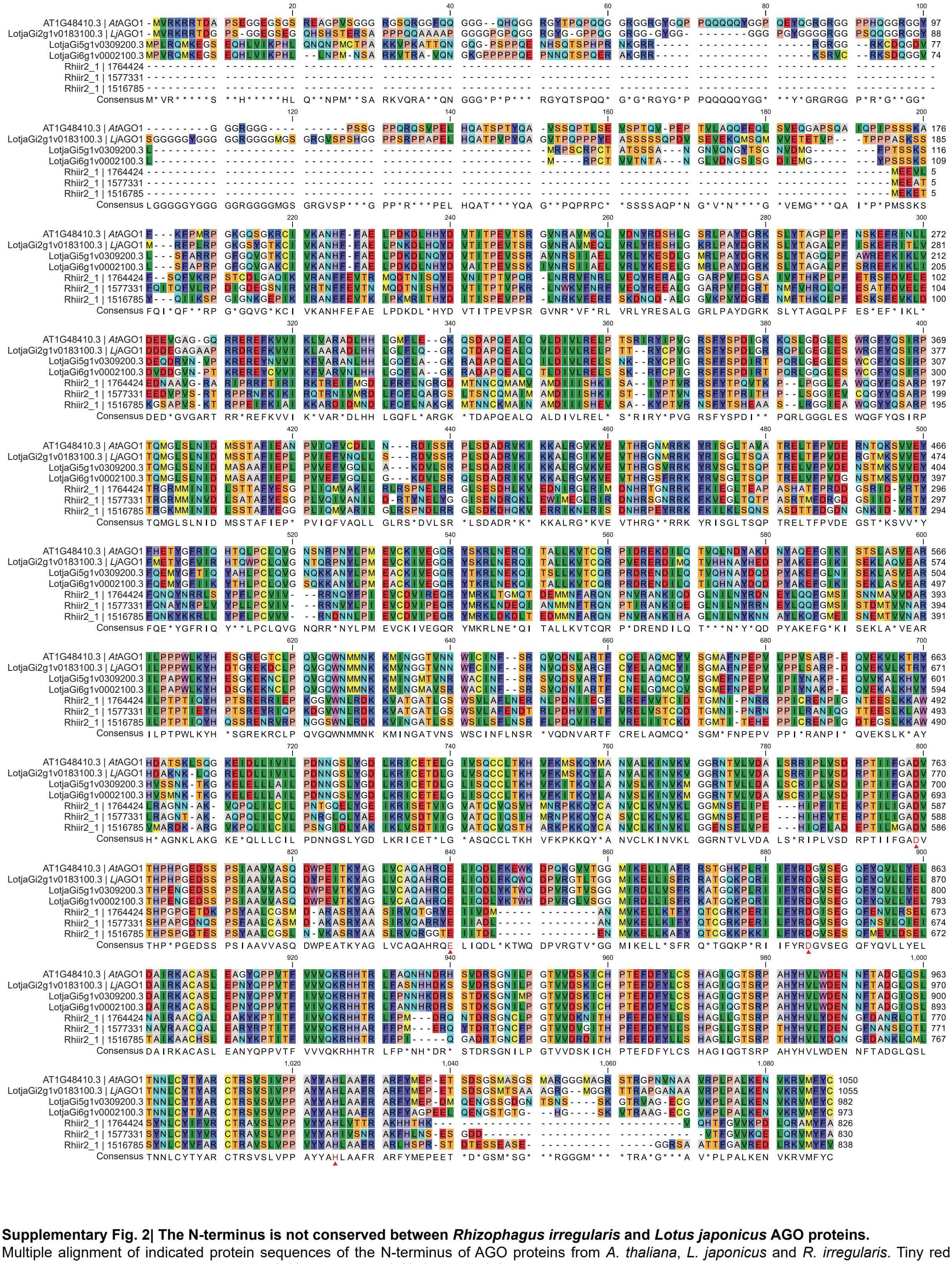

Supplementary Fig. 2| The N-terminus is not conserved between *Rhizophagus irregularis* and *Lotus japonicus* AGO proteins.
Multiple alignment of indicated protein sequences of the N-terminus of AGO proteins from *A. thaliana*, *L. japonicus* and *R. irregularis*. Tiny red triangles indicate conserved aspartic acid (D)/glutamic acid (E)/D/histidine (H) catalytic tetrad in the PIWI domain.

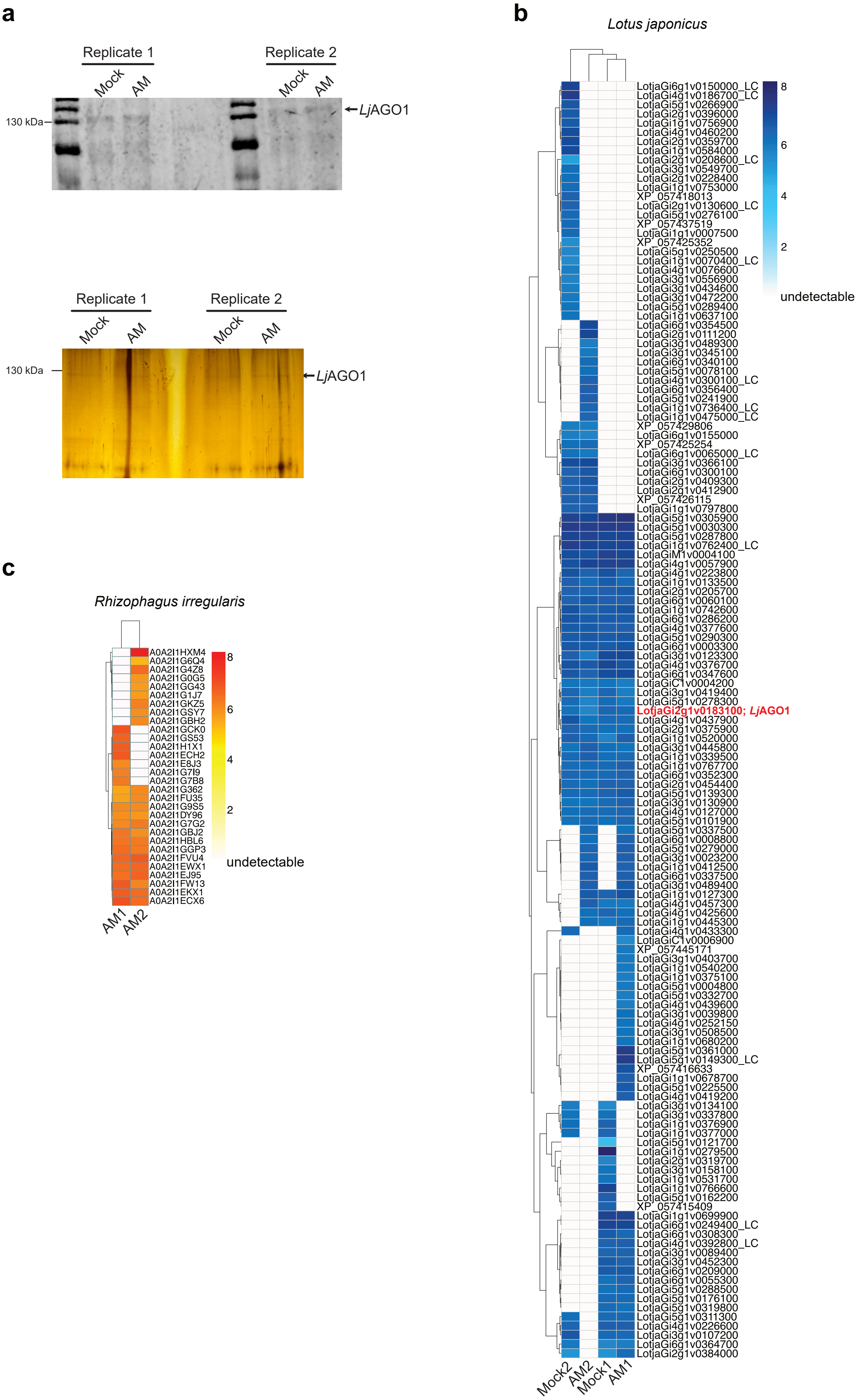
a
Replicate 1
Mock
AM
Replicate 2
Mock
AM
130 kDa
LjAGO1
Replicate 1
Mock
AM
Replicate 2
Mock
AM
130 kDa
LjAGO1
b
Lotus japonicus
LotjaGi6g1v0150000_LC
LotjaGi4g1v0186700_LC
LotjaGi5g1v0266900
LotjaGi2g1v0396000
LotjaGi1g1v0756900
LotjaGi4g1v0460200
LotjaGi2g1v0359700
LotjaGi1g1v0584000
LotjaGi2g1v0208600_LC
LotjaGi3g1v0549700
LotjaGi2g1v0228400
LotjaGi1g1v0753000
XP_057418013
LotjaGi2g1v0130600_LC
LotjaGi5g1v0276100
XP_057437519
LotjaGi1g1v0007500
XP_057425352
LotjaGi5g1v0250500
LotjaGi1g1v0070400_LC
LotjaGi4g1v0076600
LotjaGi3g1v0556900
LotjaGi3g1v0434600
LotjaGi3g1v0472200
LotjaGi5g1v0289400
LotjaGi1g1v0637100
LotjaGi6g1v0354500
LotjaGi2g1v0111200
LotjaGi3g1v0489300
LotjaGi3g1v0345100
LotjaGi6g1v0340100
LotjaGi5g1v0078100
LotjaGi4g1v0300100_LC
LotjaGi6g1v0356400
LotjaGi5g1v0241900
LotjaGi1g1v0736400_LC
LotjaGi1g1v0475000_LC
XP_057429806
LotjaGi6g1v0155000
XP_057425254
LotjaGi6g1v0065000_LC
LotjaGi3g1v0366100
LotjaGi6g1v0300100
LotjaGi2g1v0409300
LotjaGi2g1v0412900
XP_057426115
LotjaGi1g1v0797800
LotjaGi5g1v0305900
LotjaGi5g1v0030300
LotjaGi5g1v0287800
LotjaGi1g1v0762400_LC
LotjaGiM1v0004100
LotjaGi4g1v0057900
LotjaGi4g1v0223800
LotjaGi1g1v0133500
LotjaGi2g1v0205700
LotjaGi6g1v0060100
LotjaGi1g1v0742600
LotjaGi6g1v0286200
LotjaGi4g1v0377600
LotjaGi5g1v0290300
LotjaGi6g1v0003300
LotjaGi3g1v0123300
LotjaGi4g1v0376700
LotjaGi6g1v0347600
LotjaGiC1v0004200
LotjaGi3g1v0419400
LotjaGi5g1v0278300
LotjaGi2g1v0183100; LjAGO1
LotjaGi4g1v0437900
LotjaGi2g1v0375900
LotjaGi1g1v0520000
LotjaGi3g1v0445800
LotjaGi1g1v0339500
LotjaGi1g1v0767700
LotjaGi6g1v0352300
LotjaGi2g1v0454400
LotjaGi5g1v0139300
LotjaGi3g1v0130900
LotjaGi4g1v0127000
LotjaGi5g1v0101900
LotjaGi5g1v0337500
LotjaGi6g1v0008800
LotjaGi5g1v0279000
LotjaGi3g1v0023200
LotjaGi1g1v0412500
LotjaGi6g1v0337500
LotjaGi3g1v0489400
LotjaGi1g1v0127300
LotjaGi4g1v0457300
LotjaGi4g1v0425600
LotjaGi1g1v0445300
LotjaGi4g1v0433300
LotjaGiC1v0006900
XP_057445171
LotjaGi3g1v0403700
LotjaGi1g1v0540200
LotjaGi1g1v0375100
LotjaGi5g1v0004800
LotjaGi5g1v0332700
LotjaGi4g1v0439600
LotjaGi3g1v0039800
LotjaGi4g1v0252150
LotjaGi3g1v0508500
LotjaGi1g1v0680200
LotjaGi5g1v0361000
LotjaGi5g1v0149300_LC
XP_057416633
LotjaGi1g1v0678700
LotjaGi5g1v0225500
LotjaGi4g1v0419200
LotjaGi3g1v0134100
LotjaGi3g1v0337800
LotjaGi1g1v0376900
LotjaGi1g1v0377000
LotjaGi5g1v0121700
LotjaGi1g1v0279500
LotjaGi2g1v0319700
LotjaGi3g1v0158100
LotjaGi1g1v0531700
LotjaGi1g1v0766600
LotjaGi5g1v0162200
XP_057415409
LotjaGi1g1v0699900
LotjaGi6g1v0249400_LC
LotjaGi6g1v0308300
LotjaGi4g1v0392800_LC
LotjaGi3g1v0089400
LotjaGi3g1v0452300
LotjaGi6g1v0209000
LotjaGi6g1v0055300
LotjaGi5g1v0288500
LotjaGi5g1v0176100
LotjaGi5g1v0319800
LotjaGi5g1v0311300
LotjaGi4g1v0226600
LotjaGi3g1v0107200
LotjaGi6g1v0364700
LotjaGi2g1v0384000
8
6
4
2
undetectable
Mock2
AM2
Mock1
AM1
c
Rhizophagus irregularis
A0A2I1HXM4
A0A2I1G6Q4
A0A2I1G4Z8
A0A2I1G0G5
A0A2I1GG43
A0A2I1G1J7
A0A2I1GKZ5
A0A2I1GSY7
A0A2I1GBH2
A0A2I1GCK0
A0A2I1GS53
A0A2I1H1X1
A0A2I1ECH2
A0A2I1E8J3
A0A2I1G7I9
A0A2I1G7B8
A0A2I1G362
A0A2I1FU35
A0A2I1G9S5
A0A2I1DY96
A0A2I1G7G2
A0A2I1GBJ2
A0A2I1HBL6
A0A2I1GGP3
A0A2I1FVU4
A0A2I1EWX1
A0A2I1EJ95
A0A2I1FW13
A0A2I1EKX1
A0A2I1ECX6
8
6
4
2
undetectable
AM1
AM2

Supplementary Fig. 3| *Lj*AGO co-immunoprecipitation to probe for antibody cross-reactivity.
a, Immunoblot (top) and silver stain of polyacrylamide gel (bottom) of AGO1 immunoprecipitation for mass spectrometry analysis (MS). **b,** Heatmap of log_{10} intensity-based absolute quantification (iBAQ) for all 139 *L. japonicus* proteins from MS analysis. *Lj*AGO1 is labelled in red. Two replicates were conducted for which plants were independently grown and the samples were independently processed. **c,** Heatmap of log_{10}IBAQ for all 30 *R. irregularis* proteins. **a-c,** Two independent replicates were conducted.

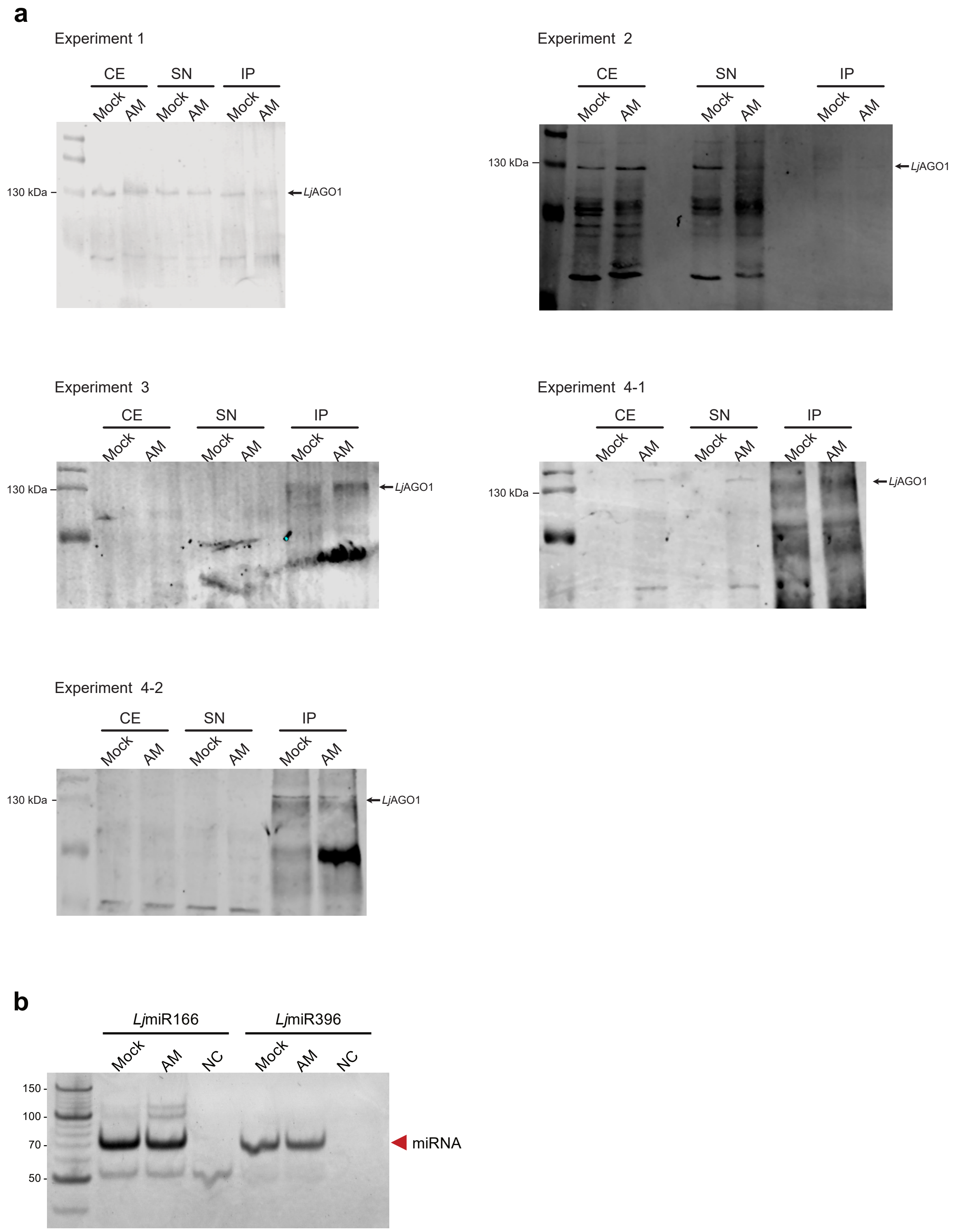

Supplementary Fig. 4| Immunoblots after *Lj*AGO1 pull-down for sequencing of associated sRNAs.
a, Immunoblot of AGO1 immunoprecipitation for sRNA sequencing using *L. japonicus* roots colonized with the fungus *R. irregularis* (AM) or without the fungus (Mock). Detection of *Lj*AGO1 using an *At*AGO1-specific antibody at the expected size of ~ 130 kDa in four independent experiments and two biological replicates in experiment four. The broad range pre-stained protein marker was used to estimate protein size. **b,** Stem-loop RT-PCR of Mock and AM *Lj*AGO1 co-IPed RNA samples to detect two *L. japonicus* miRNAs, *Lj*miR166 and *Lj*miR396. Amplicons of the expected size of 69 base pairs were visible for both miRNAs in both samples. As a negative control (NC), stem-loop RT-PCR was performed with water. The 10 bp O'Range DNA ruler (Thermo Scientific™) was used as a size marker.

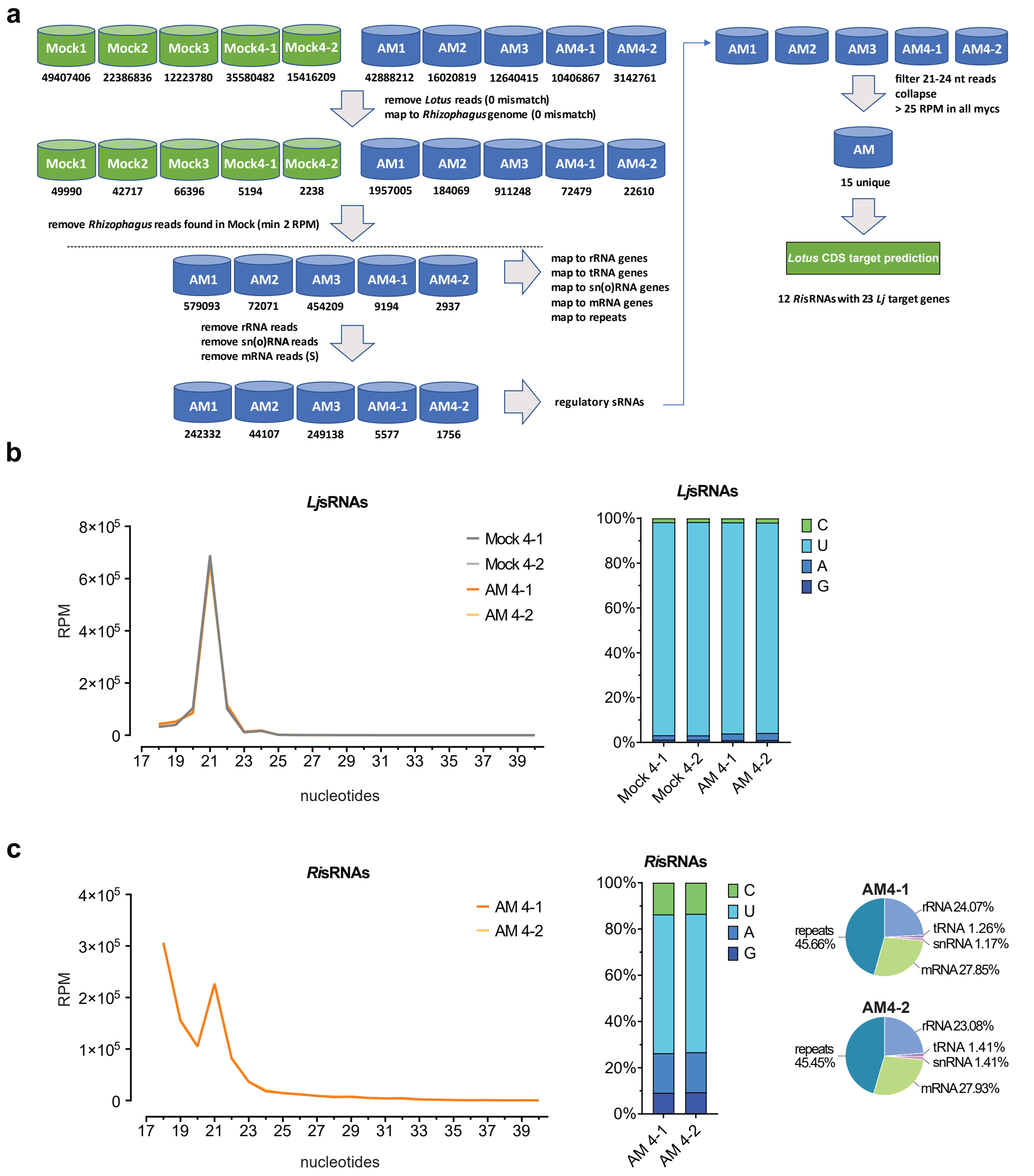

Supplementary Fig. 5| Workflow of sRNA analysis and size distribution of sRNAs in two replicates grown and processed in parallel.
a, Schematic workflow of the bioinformatic analysis of the *Lj*AGO1 co-IP small RNA NGS data and target gene prediction. Numbers below colored barrels indicate numbers of sRNAs resulting from each step of the analysis. **b,** Size profile and 5' terminal nucleotide distribution of *Lj*AGO1-associated *Lj*sRNAs of two biological replicates for which the plants were grown and the samples processed for co-IP sRNA sequencing in parallel. **c,** Size profile and 5' terminal nucleotide distribution of *Lj*AGO1-associated *Ris*RNAs of the samples shown in b. Pie charts show relative read count of *Lj*AGO-associated *Ris*RNAs mapped to distinct regions of the *R. irregularis* genome.

a

*Ris*RNA	Predicted target gene	Description
*Ris*RNA8	*LotjaGi1g1v0192600*	flavin-containing monooxygenase FMO GS-OX-like 9
	LotjaGi5g1v0238900	ABC transporter G family member 7-like
	LotjaGi5g1v0224400	heavy metal-associated isoprenylated plant protein 35-like
	LotjaGi3g1v0451000	uncharacterized protein
*Ris*RNA11	*LotjaGi3g1v0422400*	disease resistance protein RPV1-like
*Ris*RNA15	*LotjaGi1g1v0285700*	pectinesterase QRT1-like
	LotjaGi1g1v0052600	early nodulin-like protein 18
*Ris*RNA23	*LotjaGi1g1v0792000*	cyclic nucleotide-gated ion channel 1-like
	LotjaGi4g1v0232500	phosphoenolpyruvate/phosphate translocator 1, chloroplastic-like
	LotjaGi1g1v0301000	protein NTM1-like 9

```
LotjaGi1g1v0192600  5'-CAGGACGGCUAUACUUUAACU
aln                    ||o|||o||o|o|||||||.|
RisRNA8             3'-GUUCUGUCGGUGUGAAAUUAA
aln                    .|oooo.ooo...o.o|oooo
Lj. target rs       5'-GACACUUAAUUGUAAGUCCGC

LotjaGi1g1v0285700  5'-AGUAGUUACAGCACCUGGUAC
aln                    |o.||||||||||||oo|||.
RisRNA15            3'-UUCUCAAUGUCGUGGGUCAUA
aln                    ||oo.oooo|.o.|.oo|.|o
Lj. target rs       5'-AAUUCGGCUACUUCGACGAAG

LotjaGi5g1v0238900  5'-CAAGAUGGUCAUACUGUAAUU
aln                    |||||oo|o||o|||.|||||
RisRNA8             3'-GUUCUGUCGGUGUGAAAUUAA

LotjaGi3g1v0451000  5'-GAAAGCAGCCACAUUUUGAUU
aln                    .||.o||||||||o|||o|||
RisRNA8             3'-GUUCUGUCGGUGUGAAAUUAA

LotjaGi1g1v0792000  5'-CCAAUUUCGACGUCUAUUUAA
aln                    ||||.|||o||o|||||||||
RisRNA23            3'-GGUUCAAGUUGUAGAUAAAUU

LotjaGi3g1v0422400  5'-ACAAGUUACAGCAUCCAAUAU
aln                    |.|||||||||||o|||.|||
RisRNA11            3'-UUUUCAAUGUCGUGGGUCAUA
aln                    o.|.oo..o|.||o|ooo.o.
Lj. target rs       5'-CUAUUGAUAACCAACUCAAGA

LotjaGi1g1v0301000  5'-CCAACUUCAGCAUCUAUUUAU
aln                    ||||.||||o||||||||||.
RisRNA23            3'-GGUUCAAGUUGUAGAUAAAUU
aln                    o|..oooo.ooo|o.ooo|..
Lj. target rs       5'-ACUUUCCAUAACUAACCGUUU

LotjaGi5g1v0224400  5'-GUUGACAGCUACACUUUGAUU
aln                    ...||||||o|||||||o|||
RisRNA8             3'-GUUCUGUCGGUGUGAAAUUAA

LotjaGi1g1v0052600  5'-GAGAGUUGAACCACCCAGUAU
aln                    o||||||o.|.||||||||||
RisRNA15            3'-UUCUCAAUGUCGUGGGUCAUA

LotjaGi4g1v0232500  5'-UCAUCUUCAACAUCUAUUUCA
aln                    o||..||||||||||||||.|
RisRNA23            3'-GGUUCAAGUUGUAGAUAAAUU
```

b

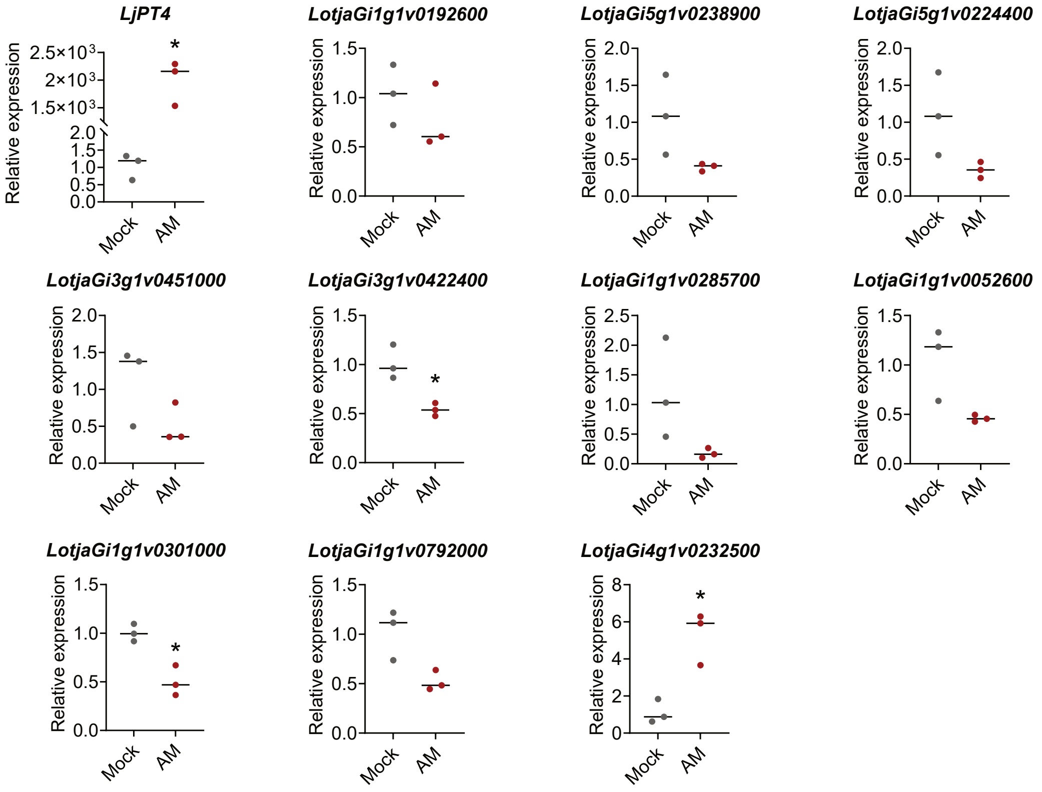

Supplementary Fig. 6| Target site alignment and expression of predicted *Ris*RNA target genes in colonised roots.
a, List of predicted target genes (top) and target site alignment (bottom) of *Ris*RNA8, *Ris*RNA11, *Ris*RNA15 and *Ris*RNA23, with predicted native target sequences in *L. japonicus* genes and the random scrambled (rs) target sequence versions, as used in the switch-on ckRNAi reporter construct and for the STTM experiment (Fig. 2, Supplementary Fig. 7). **b,** Transcript accumulation of *Ris*RNA target genes in *L. japonicus* roots colonized with *R. irregularis* (AM) or without the fungus (Mock) at 7 wpi assessed by RT-qPCR. *L. japonicus PT4* is a marker gene for root colonization by AM fungi. Expression of the housekeeping gene *Elongation Factor 1 alpha* (*EF1α*) was used for normalization. Black horizontal lines indicate medians. Asterisks indicate statistically significant differences (Kruskal-Wallis-Test; n = 3; *p_{PT4} = 0.0134, $p_{LotjaGi5g1v0238900}$ = 0.1524, $p_{LotjaGi1g1v0792000}$ = 0.0597, *$p_{LotjaGi4g1v0232500}$ = 0.0222, *$p_{LotjaGi3g1v0422400}$ = 0.0304, $p_{LotjaGi1g1v0192600}$ = 0.3635, $p_{LotjaGi1g1v0285700}$ = 0.1695, *$p_{LotjaGi1g1v0301000}$ = 0.0146, $p_{LotjaGi1g1v0288600}$ = 0.2695, $p_{LotjaGi1g1v0052600}$ = 0.1061, $p_{LotjaGi3g1v0451000}$ = 0.1812, $p_{LotjaGi5g1v0224400}$ = 0.1422).

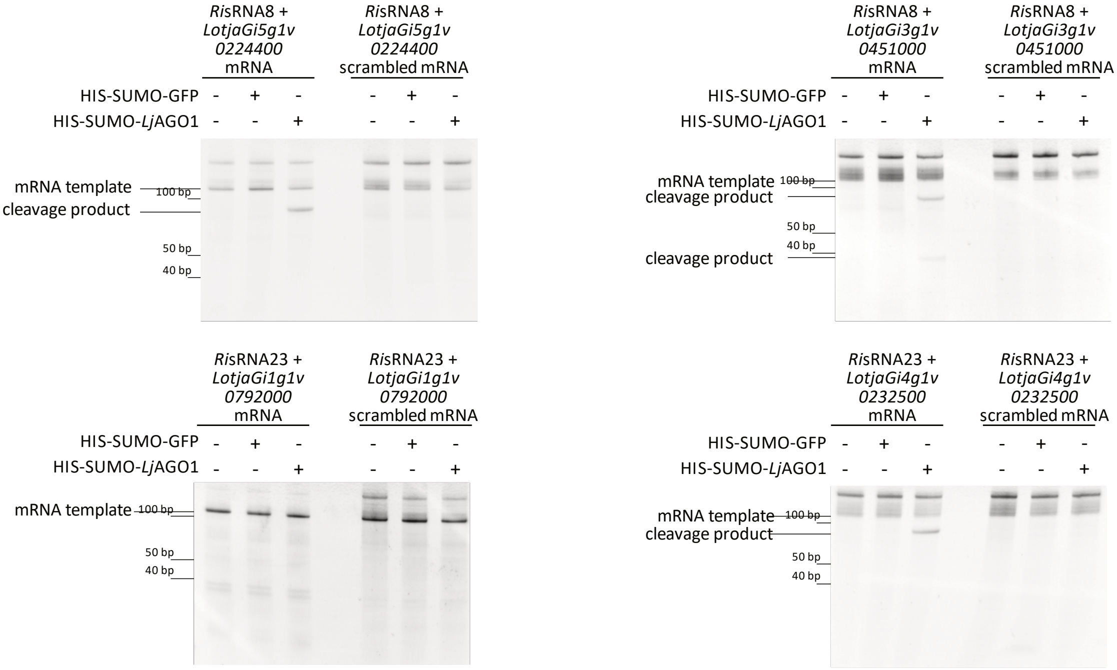

Supplementary Fig. 7| *Ris*RNAs induce target cleavage by *Lj*AGO1 *in vitro*.

In vitro cleavage assay of predicted target mRNAs by *Lj*AGO1 when bound to *Ris*RNA8 and *Ris*RNA23. The *Ris*RNA, target mRNA, and protein are listed above the gel images. The size of the mRNA template and the cleavage product are indicated on the left. Scrambled mRNA targets were included as non-target controls (right). Two independent replicates for each mRNA target/*Ris*RNA combination were conducted with similar results.

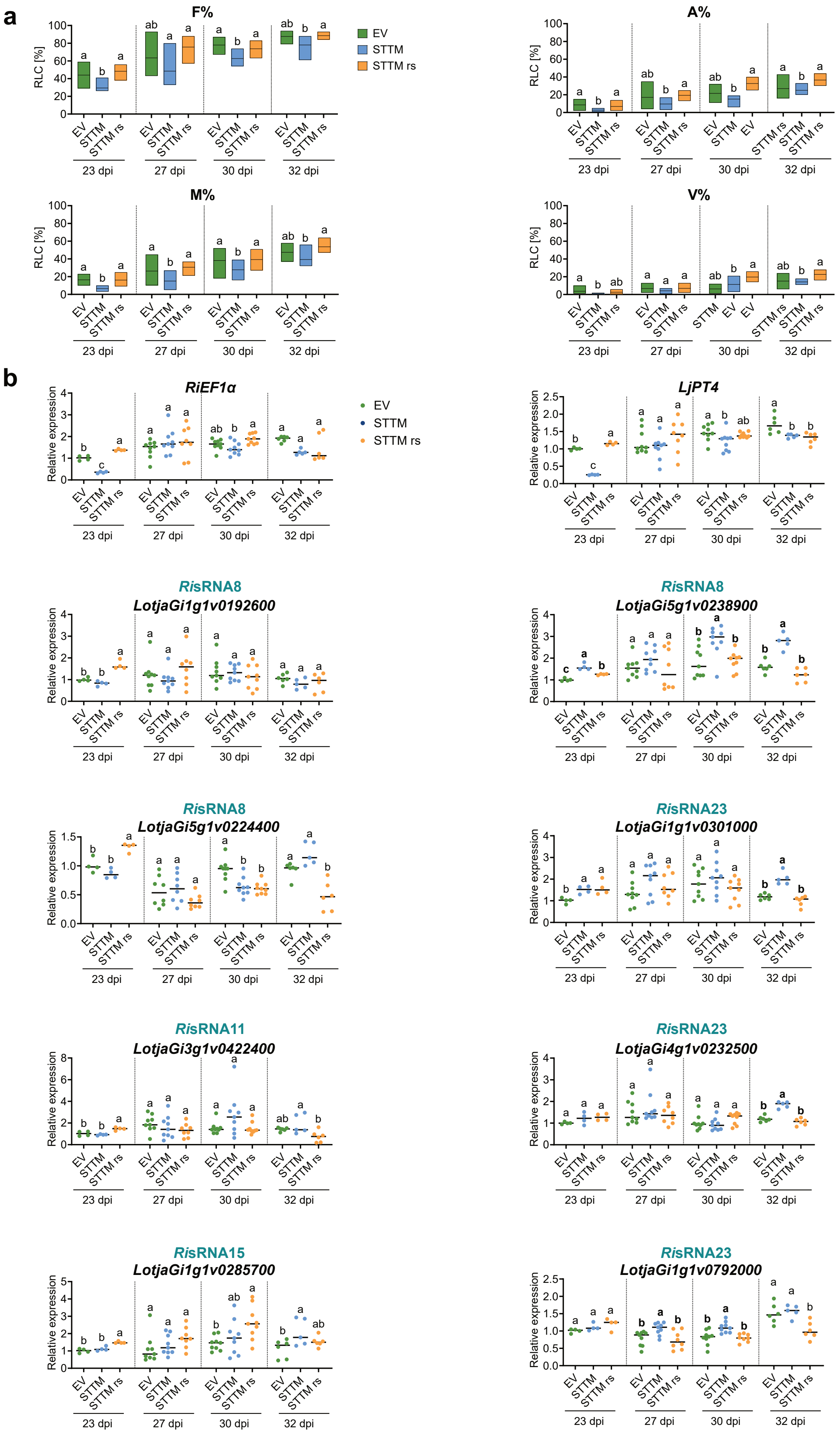

a
F%
A%
M%
V%
RLC [%]
EV
STTM
STTM rs
23 dpi
27 dpi
30 dpi
32 dpi
b
RiEF1α
LjPT4
Relative expression
RisRNA8
LotjaGi1g1v0192600
RisRNA8
LotjaGi5g1v0238900
RisRNA8
LotjaGi5g1v0224400
RisRNA23
LotjaGi1g1v0301000
RisRNA11
LotjaGi3g1v0422400
RisRNA23
LotjaGi4g1v0232500
RisRNA15
LotjaGi1g1v0285700
RisRNA23
LotjaGi1g1v0792000

Supplementary Fig. 8| Effect of STTM construct on root length colonization and expression of predicted *Ri*sRNA target genes.
a, Percent root length colonisation of *L. japonicus* hairy roots at 23, 27, 30, 32 dpi with *R. irregularis* transformed with an empty vector, an STTM construct or random scrambled STTM construct (STTM rs) as shown in Fig. 2c. at 23, 27, 30, 32 dpi with *R. irregularis*. Floating bars indicate the range (minimum to maximum) of data-points, with horizontal lines showing the mean. Different letters indicate statistically different groups (ordinary one-way ANOVA, posthoc Tukey's test; n = 6-12; $p < 0.05$). **b,** Accumulation of predicted target mRNAs for *Ri*sRNAs scavenged by the quadruple STTM construct in hairy roots from the experiment in a. The *R. irregularis* housekeeping gene *RiEF1α* and the *L. japonicus* AM marker gene *PT4* were used as markers for root colonization. Transcript accumulation was assessed by RT-qPCR and expression of the housekeeping gene *elongation factor 1 alpha* (*EF1α*) was used for normalization. *Ri*sRNAs are shown in blue above their target. Black horizontal lines indicate the median. The time course was performed once. Different letters indicate statistically different groups (ordinary one-way ANOVA, posthoc Tukey's test; n = 4-9; $p < 0.05$). The corresponding sample number and p-value are shown in: source data Fig.S8a, S8b.

a

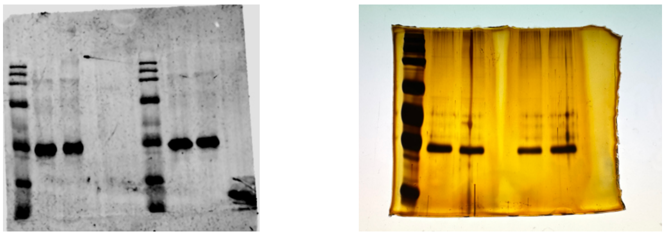

b

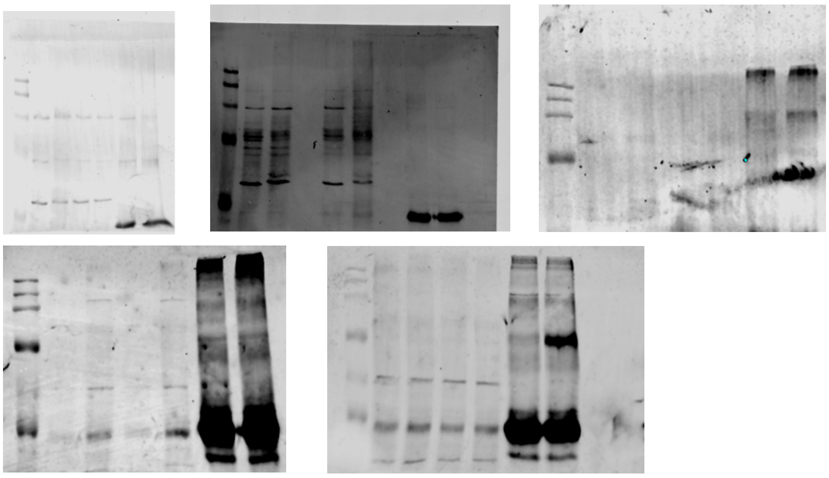

c

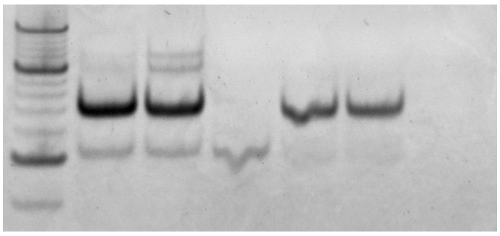

d

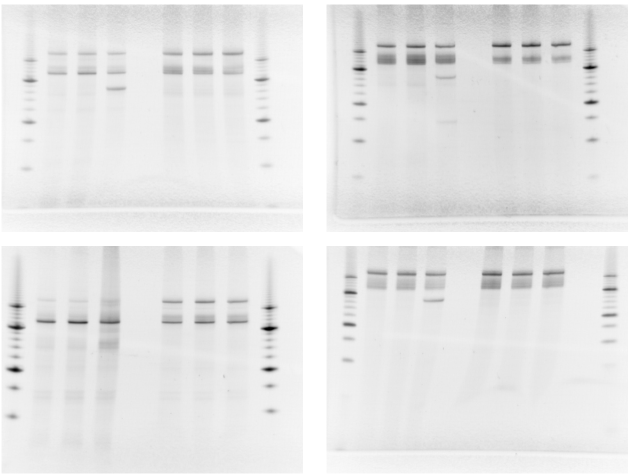

Supplementary Fig. 9| Original images of immunoblot, stem-loop PCR, and *in vitro* cleavage assay.
a, Immunoblot and silver stain of polyacrylamide gel in Fig. S3. **b,** Immunoblots in Fig. S4a. **c,** Stem-loop PCR in Fig. S4b. **d,** *In vitro* cleavage assay in Fig. S7.
