# Supplementary material for: Cross-kingdom RNA interference promotes arbuscular mycorrhiza development
Source: Nat Plants. 2026 Mar 11;12(4):695–702. doi: 10.1038/s41477-026-02247-2 (PMC13106029; doi:10.1038/s41477-026-02247-2)
Supplement: Supplementary file 4 — Sequences used for the phylogenetic tree (Supplementary Fig. 1a). [file 41477_2026_2247_MOESM4_ESM.pdf]

>AT1G48410.3 | *AtAGO1*
MVRKRRTDAPSEGGEGSGSREAGPVSGGGRGSQRGGFQQGGGQHQGGRGYT
PQPQQGGRGGRGYGQPPQQQQQYGGPQEYQGRGRGGPPHQGGRGGYGGGR
GGGPSSGPPQRQSVPELHQATSPTYQAVSSQPTLSEVSPTQVPEPTVLAQQFEQL
SVEQGAPSQAIQPIPSSSKAFKFPMRPGKGQSGKRCIVKANHFFAELPDKDLHHYD
VTITPEVTSRGVNRAVMKQLVDNYRDSHLGSRLPAYDGRKSLYTAGPLPFNSKEFR
INLLDEEVGAGGQRREREFKVVIKLVARADLHHLGMFLEGKQSDAPQEALQVLDIVL
RELPTSRIRYIPVGRSFYSPDIGKKQSLGDGLESWRGFYQSIRPTQMGLSLNIDMSS
TAFIEANPVIQFVCDLLNRDISSRPLSDADRVKIKKALRGVKVEVTHRGNMRRKYRIS
GLTAVATRELTFPVDERNTQKSVVEYFHETYGFRIQHTQLPCLQVGNSNRPNYLPM
EVCKIVEGQRYSKRLNERQITALLKVTCQRPIDREKDILQTVQLNDYAKDNYAQEFG
IKISTSLASVEARILPPPWLKYHESGREGTCLPQVGQWNMMNKKMINGGTVNNWIC
INFSRQVQDNLARTFCQELAQMCYVSGMAFNPEPVLPPVSARPEQVEKVLKTRYH
DATSKLSQGKEIDLLIVILPDNNGSLYGDLKRICETELGIVSQCCLTKHVFKMSKQYM
ANVALKINVKVGGRNTVLVDALSRRIPLVSDRPTIIFGADVTHPHPGEDSSPSIAAVV
ASQDWPEITKYAGLVCAQAHRQELIQDLFKEWKDPQKGVVTGGMIKELLIAFRRST
GHKPLRIIFYRDGVSEGQFYQVLLYELDAIRKACASLEAGYQPPVTFVVVQKRHHTR
LFAQNHNDRHSVDRSGNILPGTVVDSKICHPTEFDFYLCSHAGIQGTSRPAHYHVL
WDENNFTADGLQSLTNNLCYTYARCTRSVSIVPPAYYAHLAAFRARFYMEPETSDS
GSMASGSMARGGGMAGRSTRGPNVNAAVRPLPALKENVKRVMFYC*

>AT1G31280.1 | *AtAGO2*
MERGGYRGGRGDGRGRGGRGYGGGGGGGEQGRDRGYGGGEQGRGRGSERG
GGNRGQGRGEQQDFRSQSQRGPPPGHGGRGTTQFQQPRPQVAPQPSQAPASY
AGSVGGVAGRGAWGRKPQVPSDSASPSTSTTVVSEPVRVAEVMNLKPSVQVATS
DRKEPMKRPDRGGVVAVRRVNLYVNHYKVNFNPESVIRHYDVEIKGEIPTKKVSRF
ELAMVRDKVFTDNPDEFPLAMTAYDGQKNIFSAVELPTGSYKVEYPKTEEMRGRS
YTFTIKQVNVLKLGDLKEYMTGRSSFNPRDVLQGMDVVMKEHPSKCMITVGKSFFT
RETEPDEDFRFGVIAAKGYRHTLKPTAQGLSLCLDYSVLAFRKAMSVIEYLKLYFNW
SDMRQFRRRDVEEELIGLKVTVNHRKNKQKLTIVGLSMQNTKDIKFDLIDQEGNEPP
RKTSIVEYFRIKYGRHIVHKDIPCLDLGKNGRQNFVPMEFCDLVEGQIYPKDNLDKD
SALWLKKLSLVNPQQRQRNIDKMIKARNGPSGGEIIGNFGLKVDTNMTPVEGRVLK
APSLKLAERGRVVREEPNPRQNNQWNLMKKGVTRGSIVKHWAVLDFTASERFNK
MPNDFVDNLIDRCWRLGMQMEAPIVYKTSRMETLSNGNAIEELLRSVIDEASRKHG
GARPTLVLCAMSRKDDGYKTLKWIAETKLGLVTQCFLTGPATKGGDQYRANLALK
MNAKVGGSNVELMDTFSFFKKEDEVMFIGADVNHPAARDKMSPSIVAVVGTLNWP
EANRYAARVIAQPHRKEEIQGFGDACLELVKAHVQATGKRPNKIVIFRDGVSDAQF
DMVLNVELLDVKLTFEKNGYNPKITVIVAQKRHQTRFFPATNNDGSDKGNVPSGTV
VDTKVIHPYEYDFYLCSHHGGIGTSKPTHYYTLWDELGFTSDQVQKLIFEMCFTFTR
CTKPVSLVPPVYYADMVAFRGRMYHEASSREKNFKQPRGASTSAASLASSLSSLTI
EDKAIFKLHAELENVMFFV*

>AT1G31290.1 | *AtAGO3*
MDRGGYRGGRGDGRGRGGGGDRGRGYSGRGDGRGRGGDGDRGYSGRGDGH
GRGGGGDRGRGYSGRGDGRGRGGGGDRGRGYSGRGDGHGRGGGGDRGRGY
SGRGRGFVQDRDGGWVNPGQSSGGHVRGRGTQLQQPPPQEVPPSSSQAQVSQ
GVAPGDVGQGGVGDVGRDGVGDVGRDGVGDVGQGGVGDVGQVGVGDVGQGG
VGDVGQGGVGDVGRDGVGDVGRDGVGDVGRGGVGDRGQSQSGLSSGHFGRG
TQLQQPQPQAVSQSSSQGQVSQSFATGGVGLGAWARKPQLFSDSTVLPSSSSSN
VVASHTASGSQVMTPKPSSSDKKEPVKRPDKGGNIKVKGVINLSVNHFRVSFSTES

VIRHYDVDIKGENSSKKISRFELAMVKEKLFKDNNDFPNAMTAYDGQKNIFSAVELP
TGSFKVDFSETEEIMRGRSYTFIIKQVKELKLLDLQAYIDGRSTFIPRDVLQGMDVVM
KEHPSKRMITVGKRFFSTRLEIDFGYGVGAAKGFHHTLKPTVQGLSLCLNSSLLAFR
KAISVIEYLKLYFGWRNIRQFKNCRPDDVVQELIGLKVTVDHRKTKQKFIIMGLSKDD
TKDIKFDFIDHAGNQPPRKISIVEYFKEKYGRDIDHKDIPCLNLGKKGRENFVPMEFC
NLVEGQIFPKEKLYRDSAAWLKELSLVTPQQRLENINKMIKSSDGPRGGDIIGNFGL
RVDPNMTTVEGRVLEAPTLKLTDRRGNPIHEKLMSESNQWNLTTKGVTKGSIIKHW
AVLDFTASESLKKKMPGYFVNKLIERCKGLGMQMEAPIVCKTSSMETLYDGNALEE
LLRSVIDEASHNHGGACPTLVLCAMTGKHDGYKTLKWIAETKLGLVTQCFLTISAIK
GETVSDQYLANLALKINAKVGGTNVELVDNIFSFFKKEDKVMFIGADVNHPAAHDN
MSPSIVAVVGTLNWPEANRYAARVKAQSHRKEEIQGFGETCWELIEAHSQAPEKR
PNKIVIFRDGVSDGQFDMVLNVELQNVKDVFAKVGYNPQITVIVAQKRHQTRFFPAT
TSKDGRAKGNVPSGTVVDTTIIHPFEYDFYLCSQHGAIGTSKPTHYYVLSDEIGFNS
NQIQKLIFDLCFTFTRCTKPVALVPPVSYADKAASRGRVYYEASLMKKNSKQSRGA
SSSSASVASSSSSSVTMEDKEIFKVHAGIENFMFFV*

>AT2G27040.1 | *AtAGO4*
MDSTNGNGADLESANGANGSGVTEALPPPPPVIPPNVEPVRVKTELAEKKGPVRV
PMARKGFGTRGQKIPLLTNHFKVDVANLQGHFFHYSVALFYDDGRPVEQKGVGRK
ILDKVHQTYHSDLDGKEFAYDGEKTLFTYGALPSNKMDFSVVLEEVSATRANGNGS
PNGNESPSDGDRKRLRRPNRSKNFRVEISYAAKIPLQALANAMRGQESENSQEAIR
VLDIILRQHAARQGCLLVRQSFFHNDPTNCEPVGGNILGCRGFHSSFRTTQGGMSL
NMDVTTTMIIKPGPVVDFLIANQNARDPYSIDWSKAKRTLKNLRVKVSPSGQEFKIT
GLSDKPCREQTFELKKRNPNENGEFETTEVTVADYFRDTRHIDLQYSADLPCINVG
KPKRPTYIPLELCALVPLQRYTKALTTFQRSALVEKSRQKPQERMTVLSKALKVSNY
DAEPLLRSCGISISSNFTQVEGRVLPAPKLKMGCGSETFPRNGRWNFNNKEFVEPT
KIQRWVVVNFSARCNVRQVVDDLIKIGGSKGIEIASPFQVFEEGNQFRRAPPMIRVE
NMFKDIQSKLPGVPQFILCVLPDKKNSDLYGPWKKKNLTEFGIVTQCMAPTRQPND
QYLTNLLLKINAKLGGLNSMLSVERTPAFTVISKVPTIILGMDVSHGSPGQSDVPSIA
AVVSSREWPLISKYRASVRTQPSKAEMIESLVKKNGTEDDGIIKELLVDFYTSSNKR
KPEHIIIFRDGVSESQFNQVLNIELDQIIEACKLLDANWNPKFLLLVAQKNHHTKFFQP
TSPENVPPGTIIDNKICHPKNNDFYLCAHAGMIGTTRPTHYHVLYDEIGFSADELQEL
VHSLSYVYQRSTSAISVVAPICYAHLAAAQLGTFMKFEDQSETSSSHGGITAPGPIS
VAQLPRLKDNVANSMFFC*

>AT2G27880.1 | *AtAGO5*
MSNRGGGGHGGASRGRGGGRRSDQRQDQSSGQVAWPGLQQSYGGRGGSVSA
GRGRGNVGRGENTGDLTATQVPVASAVSGGRGRGNIGDPTFSVASSSKTVSVAS
SSKEESKNTEVSETMSNLQITSTETKPEMTSLPPASSKAVTFPVRPGRGTLGKKVM
VRANHFLVQVADRDLYHYDVSINPEVISKTVNRNVMKLLVKNYKDSHLGGKSPAYD
GRKSLYTAGPLPFDSKEFVVNLAEKRADGSSGKDRPFKVAVKNVTSTDLYQLQQFL
DRKQREAPYDTIQVLDVVLRDKPSNDYVSVGRSFFHTSLGKDARDGRGELGDGIE
YWRGYFQSLRLTQMGLSLNIDVSARSFYEPIVVTDFISKFLNIRDLNRPLRDSDRLK
VKKVLRTLKVKLLHWNGTKSAKISGISSLPIRELRFTLEDKSEKTVVQYFAEKYNYRV
KYQALPAIQTGSDTRPVYLPMELCQIDEGQRYTKRLNEKQVTALLKATCQRPPDRE
NSIKNLVVKNNYNDDLSKEFGMSVTTQLASIEARVLPPPMLKYHDSGKEKMVNPRL
GQWNMIDKKMVNGAKVTSWTCVSFSTRIDRGLPQEFCKQLIGMCVSKGMEFKPQ
PAIPFISCPPEHIEEALLDIHKRAPGLQLLIVILPDVTGSYGKIKRICETELGIVSQCCQP
RQVNKLNKQYMENVALKINVKTGGRNTVLNDAIRRNIPLITDRPTIIMGADVTHPQP
GEDSSPSIAAVVASMDWPEINKYRGLVSAQAHREEIIQDLYKLVQDPQRGLVHSGLI

REHFIAFRRATGQIPQRIIFYRDGVSEGQFSQVLLHEMTAIRKACNSLQENYVPRVT
FVIVQKRHHTRLFPEQHGNRDMTDKSGNIQPGTVVDTKICHPNEFDFYLNSHAGIQ
GTSRPAHYHVLLDENGFTADQLQMLTNNLCYTYARCTKSVSIVPPAYYAHLAAFRA
RYYMESEMSDGGSSRSRSSTTGVGQVISQLPAIKDNVKEVMFYC*

>AT2G32940.1 | *AtAGO6*
METSSSLPLSPISIEPEQPSHRDYDITTRRGVGTTGNPIELCTNHFNVSVRQPDVVF
YQYTVSITTENGDAVDGTGISRKLMDQLFKTYSSDLDGKRLAYDGEKTLYTVGPLP
QNEFDFLVIVEGSFSKRDCGVSDGGSSSGTCKRSKRSFLPRSYKVQIHYAAEIPLKT
VLGTQRGAYTPDKSAQDALRVLDIVLRQQAAERGCLLVRQAFFHSDGHPMKVGGG
VIGIRGLHSSFRPTHGGLSLNIDVSTTMILEPGPVIEFLKANQSVETPRQIDWIKAAK
MLKHMRVKATHRNMEFKIIGLSSKPCNQQLFSMKIKDGEREVPIREITVYDYFKQTY
TEPISSAYFPCLDVGKPDRPNYLPLEFCNLVSLQRYTKPLSGRQRVLLVESSRQKPL
ERIKTLNDAMHTYCYDKDPFLAGCGISIEKEMTQVEGRVLKPPMLKFGKNEDFQPC
NGRWNFNNKMLLEPRAIKSWAIVNFSFPCDSSHISRELISCGMRKGIEIDRPFALVE
EDPQYKKAGPVERVEKMIATMKLKFPDPPHFILCILPERKTSDIYGPWKKICLTEEGI
HTQCICPIKISDQYLTNVLLKINSKLGGINSLLGIEYSYNIPLINKIPTLILGMDVSHGPP
GRADVPSVAAVVGSKCWPLISRYRAAVRTQSPRLEMIDSLFQPIENTEKGDNGIMN
ELFVEFYRTSRARKPKQIIIFRDGVSESQFEQVLKIEVDQIIKAYQRLGESDVPKFTVI
VAQKNHHTKLFQAKGPENVPAGTVVDTKIVHPTNYDFYMCAHAGKIGTSRPAHYH
VLLDEIGFSPDDLQNLIHSLSYVNQRSTTATSIVAPVRYAHLAAAQVAQFTKFEGISE
DGKVPELPRLHENVEGNMFFC*

>AT1G69440.1 | *AtAGO7*
MEEKTHHHHHSTNKHIPSSKSRTPLLHKPYHHHVQTNPPPFLLHPSSHQNLNLVAS
NLPSSYYYYYYCYFYSQFHNSLPPPPPPHLLPLSPPLPPLLPLPPPHSMTRFHKSLP
VSQVVERKQQHQQKKKIQVSNNKVSGSIAIEEAALVVAKRPDFGGQDGSVIYLLAN
HFLVKFDSSQRIYHYNVEISPQPSKEIARMIKQKLVETDRNSFSGVVPAFDGRQNIY
SPVEFQGDRLEFFVNLPIPSCKAVMNYGDLREKQPQKKIEKLFRVNMKLVSKFDGK
EQRKEGEDWAPLPPEYIHALDVILRENPMEKCTSIGRSFYSSSMGGSKEIGGGAVG
LRGFFQSLRHTQQGLALNMDLSITAFHESIGVIAYLQKRLEFLTDLPRNKGRELSLEE
KREVEKALKNIRVFVCHRETVQRYRVYGLTEEITENIWFPDREGKYLRLMSYFKDH
YGYEIQFKNLPCLQISRARPCYLPMELCMICEGQKFLGKLSDDQAAKIMKMGCQKP
NERKAIIDKVMTGSVGPSSGNQTREFNLEVSREMTLLKGRILQPPKLKLDRPRNLKE
SKVFKGTRIERWALMSIGGSSDQKSTIPKFINELTQKCEHLGVFLSKNTLSSTFFEPS
HILNNISLLESKLKEIQRAASNNLQLIICVMEKKHKGYGDLKRISETRIGVVTQCCLYP
NITKLSSQFVSNLALKINAKIGGSMTELYNSIPSHIPRLLRPDEPVIFMGADVTHPHPF
DDCSPSVAAVVGSINWPEANRYVSRMRSQTHRQEIIQDLDLMVKELLDDFYKAVKK
LPNRIIFFRDGVSETQFKKVLQEELQSIKTACSKFQDYNPSITFAVVQKRHHTRLFRC
DPDHENIPPGTVVDTVITHPKEFDFYLCSHLGVKGTSRPTHYHILWDENEFTSDELQ
RLVYNLCYTFVRCTKPISIVPPAYYAHLAAYRGRLYIERSSESNGGSMNPSSVSRVG
PPKTIPLPKLSDNVKNLMFYC*

>AT5G21030.1 | *AtAGO8*
MDTTLPPPQHMEREPLKSKSSLLPMTRRGNGSKGQKILLLTNHFRVNFRKPNSHN
FFHYSVTITYEDGSPLLAKGFGRKILEKVQQTCQADLGCKHFAYDGDKNLYTVGPL
PRSSLDFSVVLETAPSRRNADKRLKLPHQSKKFNVAILFAPPEIPMEAIANALQGKK
TKHLLDAIRVMDCILSQNAARQGCLLVRQSFFHNDAKYFANIGEGVDCCKGFHSSF
RTTQGGLSLNIDVSTAMIVKPGPVVDFLIANQGVNDPFSINWKKAKNTLKNLRVKVL
PSNQEYKITGLSGLHCKDQTFTWKKRNQNREFEEVEITVSDYFTRIREIELRYSGGL

PCINVGKPNRPTYFPIELCELVSLQRYTKALTKFQRSNLIKESRQNPQQRIGVLTRAL
KTSNYNDDPMLQECGVRIGSDFTQVEGRVLPTPKLKAGKEQDIYPINGSWNFKNKP
ATVTRWAVVNFSARCDPQKIIDDLTRCGKMKGINVDSPYHVVFEENPQFKDATGSV
RVDKMFQHLQSILGEVPPKFLLCILEKKNSDVYEKSCSMWNCECIVPPQNLNDQYL
TNLLLKINAKLGGLNSVLDMELSGTMPLVMRVPTIIIGMDVSHGSPGQSDHIPSIAAV
VSSREWPLISKYRACVRTQSPKVEMIDSLFKPVSDKDDQGIMRELLLDFHSSSGKK
PNHIIIFRDGVSESQFNQVLNIELDQMMQINHHTKFFQTESPNNVLPGTIIDSNICHQ
HNNDFYLCAHAGKIGTTRPTHYHVLYDEIGFDTDQLQELVHSLSYVYQRSTTAISLV
APICYAHLAAAQMATAMKFEDMSETSSSHGGITTAGAVPVPPMPKLNTNVASSMFF
C*

>AT5G21150.1 | *AtAGO9*
MDSDEPNGSGLPPPPPFVPANLVPEVEPVKKNILLPMARPRGSGSKGQKIPLLTNH
FGVKFNKPSGYFFHYSVAINYEDGRPVEAKGIGRKILDKVQETYQSDLGAKYFAYD
GEKTLFTVGALPSNKLDFSVVLEEIPSSRNHAGNDTNDADRKRSRRPNQTKKFMVE
ISYAAKIPMQAIASALQGKETENLQDALRVLDIILRQSAARQGCLLVRQSFFHNDVKN
FVPIGGGVSGCRGFHSSFRTTQGGLSLNIDTSTTMIVQPGPVVDFLLANQNKKDPY
GMDWNKARRVLKNLRVQITLSNREYKISGLSEHSCKDQLFTWRKPNDKGEFEEVEI
TVLNYYKERNIEVRYSGDFPCINVGKPKRPTYFPIEFCNLVSLQRYTKSLTNFQRAA
LVEKSRQKPPERMASLTKGLKDSNYNADPVLQDSGVSIITNFTQVEGRILPTPMLKV
GKGENLSPIKGKWNFMRKTLAEPTTVTRWAVVNFSARCDTNTLIRDLIKCGREKGI
NVEPPFKDVINENPQFRNAPATVRVENMFEQIKSKLPKPPLFLLCILAERKNSDVYG
PWKKKNLVDLGIVTQCIAPTRLNDQYLTNVLLKINAKLGGLNSLLAMERSPAMPKVT
QVPTIIVGMDVSHGSPGQSDIPSIAAVVSSRQWPLISKYKACVRTQSRKMEMIDNLF
KPVNGKDEGMFRELLLDFYYSSENRKPEHIIIFRDGVSESQFNQVLNIELDQMMQA
CKFLDDTWHPKFTVIVAQKNHHTKFFQSRGPDNVPPGTIIDSQICHPRNFDFYLCAH
AGMIGTTRPTHYHVLYDEIGFATDDLQELVHSLSYVYQRSTTAISVVAPVCYAHLAA
AQMGTVMKYEELSETSSSHGGITTPGAVPVPPMPQLHNNVSTSMFFC*

>AT5G43810.1 | *AtAGO10*
MPIRQMKDSSETHLVIKTQPLKHHNPKTVQNGKIPPPSPSPVTVTTPATVTQSQASS
PSPPSKNRSRRRNRGGRKSDQGDVCMRPSSRPRKPPPPSQTTSSAVSVATAGEI
VAVNHQMQMGVRKNSNFAPRPGFGTLGTKCIVKANHFLADLPTKDLNQYDVTITPE
VSSKSVNRAIIAELVRLYKESDLGRRLPAYDGRKSLYTAGELPFTWKEFSVKIVDED
DGIINGPKRERSYKVAIKFVARANMHHLGEFLAGKRADCPQEAVQILDIVLRELSVK
RFCPVGRSFFSPDIKTPQRLGEGLESWCGFYQSIRPTQMGLSLNIDMASAAFIEPLP
VIEFVAQLLGKDVLSKPLSDSDRVKIKKGLRGVKVEVTHRANVRRKYRVAGLTTQPT
RELMFPVDENCTMKSVIEYFQEMYGFTIQHTHLPCLQVGNQKKASYLPMEACKIVE
GQRYTKRLNEKQITALLKVTCQRPRDRENDILRTVQHNAYDQDPYAKEFGMNISEK
LASVEARILPAPWLKYHENGKEKDCLPQVGQWNMMNKKMINGMTVSRWACVNFS
RSVQENVARGFCNELGQMCEVSGMEFNPEPVIPIYSARPDQVEKALKHVYHTSMN
KTKGKELELLLAILPDNNGSLYGDLKRICETELGLISQCCLTKHVFKISKQYLANVSLK
INVKMGGRNTVLVDAISCRIPLVSDIPTIIFGADVTHPENGEESSPSIAAVVASQDWP
EVTKYAGLVCAQAHRQELIQDLYKTWQDPVRGTVSGGMIRDLLISFRKATGQKPLR
IIFYRDGVSEGQFYQVLLYELDAIRKACASLEPNYQPPVTFIVVQKRHHTRLFANNH
RDKNSTDRSGNILPGTVVDTKICHPTEFDFYLCSHAGIQGTSRPAHYHVLWDENNF
TADGIQSLTNNLCYTYARCTRSVSIVPPAYYAHLAAFRARFYLEPEIMQDNGSPGKK
NTKTTTVGDVGVKPLPALKENVKRVMFYC*

>LotjaGi2g1v0183100.3

MVRKRRTDGPSGGEGSEGQHSHSTERSAPPPQQAAAAPGGGGPGPQGGRGYG
GPPQGGRGGGYGGGGGPGYGGGGRGGPSQQGGRGGYSGGGGGYGGGGGRG
GGGMGSGRGVSPSHGGPPSRPPAPELHQATPVPYQAGVTPQPPPYEASSSSSQ
PDVSEVEKQMSQMVVETETVPTPPPASKSSMRFPLRPGKGSYGTKCIVKANHFFA
ELPNKDLHQYDVTITPEVTSRGVNRAVMEQLVRLYRESHLGKRLPAYDGRKSLYTA
GALPFISKEFRITLVDDDEGAGAAPRRDREFKVVIKLAARADLHHLGLFLQGRQTDA
PQEALQVLDIVLRELPTTRYCPVGRSFYSPDLGRRQPLGEGLESWRGFYQSIRPTQ
MGLSLNIDMSSTAFIEPLPVIEFVNQLLSRDVSSRPLSDADRVKIKKALRGIKVEVTH
RGNMRRKYRISGLTSQATRELTFPVDERGTMKSVVEYFMETYGFVIRHTQWPCLQ
VGNTQRPNYLPMEVCKIVEGQRYSKRLNERQITALLKVTCQRPVERERDIMQTVHH
NAYHEDPYAKEFGIKISEKLAQVEARILPPPWLKYHDTGREKDCLPQVGQWNMMN
KKMVNGGTVNNWFCINFSRNVQDSVARGFCYELAQMCYISGMAFNPEPVVPPLSA
RPDQVEKVLKTRYHDAKNKLQGRELDLLIVILPDNNGSLYGDLKRICETDLGLVSQC
CLTKHVFKMSKQYLANVALKINVKVGGRNTVLVDALSRRIPLVSDRPTIIFGADVTHP
HPGEDSSPSIAAVVASQDYPEITKYAGLVCAQAHRQELIQDLFKQWQDPVRGTLTG
GMIKELLISFRRATGQKPQRIIFYRDGVSEGQFYQVLLFELDAIRKACASLEPNYQPP
VTFVVVQKRHHTRLFASNHHDKSSVDRSGNILPGTVVDSKICHPTEFDFYLCSHAGI
QGTSRPAHYHVLWDENNFTADGLQSLTNNLCYTYARCTRSVSIVPPAYYAHLAAFR
ARFYMEPETSDSGSMTSAAAGRGMGGRTTRAPGANAAVRPLPALKENVKRVMFY
C

>LotjaGi5g1v0309200.3
MPLRQMKEGSEQHLVIKPHLQNQNPMCTPAKKVPKATTQNGQGPSPPQENHSQT
SPHPRNKGRRRGRGGRKCDQGDVLMRPSCRPCTATSSSANGNVQNGYTSGNVD
MGFPTSSKSLSFARRPGFGQVGTKCIVKANHFFAELPDKDLNQYDVTITPEVSSRA
VNRSIIAELVRLYKESDLGMRLPAYDGRKSLYTAGQLPFAWREFKIKLVDEQDRVNV
PKREREYNVVIKFVARANLHHLGQFLAGKRADAPQEALQILDIVLRELSNKRYCPIG
RSFFSPDIRTPQRLGEGLESWCGFYQSIRPTQMGLSLNIDMASAAFIEPLPVVEFVG
QLLAKDVLSRPLSDADRIKIKKALRGVKVEVTHRGSVRRKYRVSGLTSQPTRELVFP
VDENSTMKSVVEYFQEMYGFTIQYAHLPCLQVGNQKKANYLPMEACKIVEGQRYT
KRLNEKQITSLLKVTCQRPRDRENDILQTVQHNAYDQDPYAKEFGLNISEKLASVEA
RILPAPWLKYHESGKEKNCLPQVGQWNMMNKKMINGMTVNRWACINFSRSVQDS
VARTFCNELAQMCQVSGMEFNPEPVIPIYNAKPEQVVKALKHVYHVSSNKTKGKEL
ELLLAILPDNNGSLYGDLKRICETDLGLISQCCLTKHVFKITKQYLANVSLKINVKMGG
RNTVLLDALSCRIPLVSDIPTIIFGADVTHPENGEDSSPSIAAVVASQDWPEVTKYAG
LVCAQAHRQELIQDLYKTWQDPVRGTVSGGMIRDLLVSFRKATGQKPLRIIFYRDG
VSEGQFYQVLLYELDAIRKACASLEPNYQPPVTFIVVQKRHHTRLFPNNHKDRSST
DKSGNIMPGTVVDSKICHPTEFDFYLCSHAGIQGTSRPAHYHVLWDENNFTADGIQ
SLTNNLCYTYARCTRSVSVVPPAYYAHLAAFRARFYMEPDMQENGSSGDGNTSNS
SKGTRVAGECGVKPLPALKENVKRVMFYC

>LotjaGi2g1v0300100.1
MEETEDSTNATQKLTTKTRSFINGANSHEHHHYHQHHHLLQYSAQVGFCNNNNQN
KYQRYYPALLPLPSLIPLQQLPLIPPFPQNQSINSKTHLQKPPCKLNSSPSSDYKLSE
PALAPDSAPKELQRQTKESFKGDDGRKLIPTRKQQDLIVARRPDSGGKEGSVISLLA
NHFLVQFDPSQKIYHYNVEITPHPSKDVAREIKQKLVNNNSAMLSGALPAYDGRQN
LYSSIEFQNDKLEFYISLLIPTSKLTSPYGEMYDLKEKKEQHKLFRINIKLVSKIDGKET
NYLSKEGDEWIPLPQDYLHALDVVLRESPTEKCIPVGRSFYSNSMGRSKDIGGGAV
GLRGFFQSLRPTQQGLALNLDFSVTAFHESIGVISYLQKRLEFLRDLSQRKTTQLTC
EERKEVEKALKNIRVFVCHRETVQRYRVYGLTEEATENLWFADRDGQNLRLVNYF

KDHYNYDIQFRKLPCLQISRSKPCYLPMELCVICEGQKFLGKLSDDQTARILKMGCQ
RPGERKTIIEGVMRGNVGSTSGEQEREFKLQVSREMTKLTGRILHPPKLKLGDGGH
VRNLTPSRHDRQWNLLDGNVFEGTTIERWALVSFGGTPEQKSNIPRFINQLCQRCE
QLGIFLNKNTVMSPQFESSQVLNNVTLLESKLKRIQRTASNNLQLLICVMERKHKGY
ADLKRIAETSIGLISQCCLYPNLCKLSSQFLANLALKINAKVGGCTVALYNSLPSQLP
RLFHIDEPVIFMGADVTHPHPLDDSSPSVAAVVGSMNWPTANKYISRIRSQTHRQEI
IQDLGPMVGELLDDFYQEVEKLPNRIVFFRDGVSETQFHKVMQEELQSIRHACERF
PDYKPLITFAVVQKRHHTRLFPFPGETDPSSPQNNFLYENIPPGTVVDSVITHPKEF
DFYLCSHWGVKGTSRPTHYHVLWDENQFTSDELQKLVYNLCYTFVRCTKPISLVPP
AYYAHLAAYRGRLYLERSESLGLFRNTSTLSRAAPPKTAPLPKLSENIKKLMFYC

>LotjaGi4g1v0085500.3
MERTGYRNRGNHGNGGRGGGFAGQNQQQPGAAGGGGGNGGRGGGFQGQQQ
PVGGGGGGRGGFYGHQTGGSGGAGGRGGGDGRGGFPGQYQLGGGSGSRGG
GFHGQHYQPVGGGGASGRGGGDGRGGGFQGQYKQQRGGGGGGGRGGGFQG
QYQQQPGGGGGRGGGGRGGASDRGFQSQQQEQQLWSQKGNPSSSSVQTNITI
GSSILPSTSRTNHPNTHPDIGRLTISDDAAVEKISPVHRPDKGGTLAVRNCKLHVNH
FRVGFDSQGTIMHYDVDVKPSLPPQNGRPQKISKFDLSLIRDKLFSDDPQRLPLLKT
AYDGEKNIFSAVLLPEETFIVDVSKGEDERTISYAVTLTLVNKLPLHKLKDYISGKVLN
IPRDILQGMDLVVKENPSKRTVSLGRCFFPTEHPLIERDLEPGVIAIGGFQHSLKPTS
QGISICLDYQVLSFHKKMSVLDFLYARIQGFNIDEFWKYKKDVELSLIGLKVNVTHRR
TKQKYTIAKLTTEDTRHITFTKVDPEGQNPPTKTTLVAYFKDKHGVDITYKDIPSLVF
VGSKTNYVPMELCDLVDGQRFPKELLDKYPANNLKKMSLCRPSERESIIQKMMKSN
AGPCGGEILQNFGMTVKTSMTDVTARVIRPPTLKLGHPSGKIISEPLRPEKVHWNLV
EKSMVEGKPVECWGILDFTSKGPTRWKLRGTQFVENLLDKYNRKLGIDMKEPVWY
EHSAMWKLGDYNLLYELLEQINDKVQKKCGQRLQFLLCVMAGKDQGYKCLKWIAE
TKVGLVTQCCLSGNANEGSDQYLTNLALKINAKIGGTNVELVNRLPHIDGEGDVMFI
GADVNHPGSRDINSPSIAAVVATVNWPAANRYAARVCAQGHRVEKIVNFGEVCLDL
VTYYESLNKVRPEKIVIFRDGVSESQFLMVLTEELQDLRRAFSRSNYFPTITLIVAQK
RHQTRLFPASAKDGAPSGNVLPGTVVDTIVVHPFEFDFYLCSHYGSLGTSKPTHYH
VLWDEHKFSSDDLQKLIYDMCFTFARCTKPVSLVPPVYYADLAAYRGRLYYEAKIG
VQSPGSNSSSSSSPLASSSFSSIASYSNDASCYKLHPTMENVMFFV

>LotjaGi6g1v0275900.2
MVREKLFSDDPERLPLDMTAYDGANTIFSAVHLPEETFTVEITEGEDEKTVSYSVSI
SLVNKLRLRKLMDYLCAHTISIPRDILHGMDVVVRENPARRTISVGRHFYPSNPPLV
MKDLHHGNIAVGGFQHSLKPTSQGLSLCVDYSVLAFRKQMSVLDFLHECIDNFKLV
EFYHFRKYVEEALIGLKVNVTHRKSKQKYVIAGLTPKVTRYVTFPIDDTKGWNLSKD
VSLLSFFKDKYGKDIVYKDIPCLDLGKGNKKNYVPMEFCVLVDGQRCTREHLGGVA
ANTLKAMSLAHPNERESAIQKMVQSSDGPCGGDLIQNFGMSVNTTMTTIVGRVICP
PELKLGDPNGKNIKITVDMEKCHWNLAGRSMVEGKAIERWGILDFTSYGPYKYKLR
AKEFIQKLIGKYKKLGINMQEPIWYEESSTTILASYDLLSELLAKINNICKYNQGRLQF
LLCVMAKKSSGYKYLKWISETKIGIVTQCCLSSSANEGEDKFYTNLALKINAKLGGS
NVELSNRLPYFEGEGHVMFVGADVNHPGSRDTRSPSIAAVVATVNWPAANRYAAR
VCPQYNRCEKILNFGEVCLELVTCYCRINGVRPERIVVFRDGVSEYQFDMVLNEELL
DLKKAFQRLNYFPTITLIVAQKRHHTRFFPEGWRDGSSSGNILPGTIVDTKVTHPFE
FDFYLCSYYGSLGTSKPTHYHVLWDEHKFKSDELQKLIYEMCFTFARCTKPVSLVP
PVYYADLAAYRGRLYHEARVGMQSQKSAASSSSKDSFEQGLYRLHADLENIMFFI

>LotjaGi6g1v0190900.4

MDSFEPDGNGKEESLPLPPPPPVLPSDVVPLKAEEVLPAAPPPEPVKKKTPARLPIA
RRGLGTKGTKIPLLTNHFKVTCTNNDGHFFHYSVNFTYEDGRPVEGKGVGRKIMDK
VQETYQSDLNGKDFAYDGEKSLFTIGSLPRNKLEYEVVLEDVTSNRNNGNCSPPN
GHGEDNETDKKRMRRPYRAKTFKVEISYAAKIPMQAIANALRGQESENFQEAIRVL
DIILRQHAAKQGCLLVRQNFFHNDPKNFADVGGGVLGCRGFHSSFRTTQSGLSLNI
DVSTTMIVQPGPVVDFLISNQNVRDPFQLDWTKAKRTLKNLRIKTYPSNQEFKITGF
SELPCKEQTFSLRKKGDGDDSEEVTVYDYFVNTRKIELRYSGDLPCINVGKPKRPT
FFPIELCELVSLQRYTKALTTLQRSSLVEKSRQKPQERMNVLTDALKISNYGAEPLL
KNCGISISNGFTQVEGRVLPAPRLKFGNGEDMNPRNGRWNVARMKFVRPTAKVE
RWAVANFSARCDVQALVRDLMRIADMKGIQMDQPFDVFAESPQFRRAPPMVRVE
KMFEDIQSKLPGAPQFLLCLLPDRKNCEIYGPWKKKNLADFGIVNQCMCPLRVNDQ
YLTNIMLKINAKLGGLNSLLSVELSPSLPIVSKAPTLILGMDVSHGSPGQTDIPSIAAV
VSSREWPLISKYRACVRTQSAKVEMIDNLFKQVSEKEDEGIMRELLLDFYLSSGKR
KPDNIIIFRDGVSESQFNQVLNVELDQIMEACKFLDDKWEPKFVVIVAQKNHHTRFF
QPGSPDNVPPGTIIDNKIGHPRNYDFYLCAHAGMIGTSRPTHYHVLLDQAGFSPDE
LQELVHSLSYVYQRSTTAISVVAPICYAHLAATQLGQFMKFEDKSETSSSHGGLSAA
GAVPVPQLPKLQDNVCNSMFFV

>LotjaGi3g1v0481700.1
MSRRGGRQQPDPRRDQPFPTPSPHPDAGRGRGRGRARAPPSQQPPSSSSHSPA
PVIGAPISPAPVVSAPISAPSPPPPSPVPVDALSSEVEQKLALRPAAPSSTKAIRFPD
RPGFGRLGKKIQVRANHFQLQVAERDLHHYDVAITPEITSKKVTREVVSQLIKMYKE
SVLGNRLPVFDGRKNLFTAGPLPFSSKEFVVKLEDDRPKASSSSGSKKEKRERQFK
VTIRFAAKVDLHHLFQFLGRQQLDCPQNTIQALDVALRATASEKYNVVGRSFFSPEL
GQTGPLGSGTEYWRGYYQSLRPTQMGLSLNIDVSARAFFEPIPVTEFVPKHFRNIN
FSRDQDRVKVKKALRGIRVDVFLGECKRSYKISGVSREPVKDLMFTLDDQKTKKSV
AQYFTEKYKVTLKHANLPALQAGSDTKPIYLPMEVCVIAAGQRYTKRLNEEQVTALL
RATCQRPQDRENYIKQIVKQHNFNNDKFVREFGISVKEDPTLLNARVLPPPRLKYH
ESGKEPRVDPWMGQWNMINKKMVDGGKVEHWSCLNFSSRLRPDLPSIFCDELRS
MCTSKGMVFNPQPLVPIKTVNPLQIESALQNLHKQSITNLANMKQQGRLQLLIIILPD
VKGSYGKIKKICETELGIVSQCCQPRQVQKLNKQYLENLALKINVKVGGRNTVLSDA
FDRRIPHVSDKHTIIFGADVTHPQPGEDSSPSIAAVVASMDWPWVTKYKGTVSAQA
HREEIIQDLFTTFEDPKRGLVQGGIIRELIRSFYIANGKRKPERIIFYRDGVSEGQFSQ
VLLYEMDAIRKACMSLEDGYLPRVTFVVVQKRHHTRLFPADHRSRDQMDKSGNIM
PGTVVDTSICHPREFDFYLNSHAGIQGTSRPTHYHVLYDENNFTADELQGLTNNLC
YTYARCTRSVSIVPPAYYAHLAAFRARSYIEGESEMGSASAGAGGATRSNVEIKLPA
IKDNVKDVMFYC

>LotjaGi6g1v0023300.1
MEKVPEIEDPADAEPPPLSPSSPDVSPSIETVEADPIKFSIISRQGVGTAGKRIPVLTN
LFKVSVNAPDAIFFQYSVTIATEDKRVIESKGIGRKLIDRLYQTYSSELGGKRFAYDG
DRTLYTVGPLPQNKFEFKVLLEESFAKSSAECPDANGSPRGETKRSKCSFQSKAF
MVDISFTAKIPLQSVALSLEGMGTDANCQDALRVLDTVLRQQAANRGCLLVRQSFY
HDDSRNFSDVGGGVTGVRGFHSSFNLKQGGLSLNMDVSTTVILKPGPVIDFLLSNQ
NVKEPRYIDWAKAKKVLKNLRVLATHRKWEFKISGLSEKPCIQQLFSMKVKNGDNN
DGEQTVDITVYEYFAEHCGIELTSSAYLPCLDVGKPNRPIYLPLELCSLVPLQRYKKA
LSPVQRASLIEKSRQKPQDRIKSLRNAVEDYHYDDDPVLAACGISIDKQLTQVEARV
LETPKLKVGKNDDCIPQNGRWNFNKKTFLQPSRIDFWAVVNFSARCDTSYITRELIK
CGMSKGINIERPYTLIEEDAPMKKSNPVARVEKMFDLLTSKLIREPKLILCVLPEKKN
CDIYGPWKKKCLSEFGVVTQCISPLKITDQYLTNVLLKINSKLGGINSLLTIELSGNLP

LIEDTPTMILGMDVSHSSPGQSDVPSIAAVVGSRLWPLISRYRASVRTQSSKVEIIDA
LYKPLDGGKDDGIIRELLLDFYESSNGRKPAQIIVFRDGVSESQFNQVLDIEVNQIIKA
YQHLGEVDVPKFTVIVAQKNHHIKLFQANSVDNVPPGTVVDTKIVHPRNYDFYLCAH
AGMIGTSRPVHYNVLLDEIGFSPDSLQNLIHSLSYANQRSSIATSIVAPIHYAHHAAA
QMRQVLNFDNLSETSSNLDSEGVVSIPELPRLHSNVKSTMFFC

>LotjaGi1g1v0056000.1
MDPNEHENGNGNGNGNEDLMPPPPPPPIVPADVEPVKVDLLDLPPEPVKKKLPTR
LPIARKGLGSKGTKLPLLTNHFKVTVANSDGHFFQYSVALSYEDGRPVEGKGVGRK
VIDKVQETYGSELNGKDFAYDGEKTLFTIGSLARNKLEFTVVLEDVISNRNNGNCSP
DGASTNDSDKKRMRRPYHSKTFKVEISFAAKIPLQAIVNALRGQESENYQEAIRVLD
IILRQHAAKQGCLLVRQSFFHNDPKNYADVGGGVLGCRGFHSSFRTTQSGLSLNID
VSTTMIIQPGPVVDFLIANQNVRDPFSLDWAKAKRTLKNLRIKASPSNQEYKITGLSE
LPCKEQTFTMKKKGGNNGEEDATEEEITVYEYFVNYRKIDLRYSADLPCINVGKPKR
PTYVPVELCSLVSLQRYTKALTTLQRSSLVEKSRQKPLERMNVLNQALKTSNYGNE
PMLKNCGITIASGFTQVEGRVLQAPRLKFGNGEDFNPRNGRWNLNNKKVVRPAKIE
HWAVVNFSARCDVRGLVRDLIKCARLKGIPIDEPYEEIFEENGQFRRAPPLVRVEKM
FERIQKELPGAPSFLLCLLPERKNSDLYGPWKKKNLAEYGIVTQCISPTRVNDQYLT
NVLMKINAKLGGLNSVLGVEMNPSIPIVSKVPTIILGMDVSHGSPGQSDIPSIAAVVS
SREWPLISKYRACVRTQSPKVEMIDNLFKQVSEKEDEGIIRELLIDFYSSSGKRKPD
NIIIFRDGVSESQFNQVLNIELNQIIEACKFLDETWNPKFLVIVAQKNHHTKFFQPGSP
DNVPPGTVIDNKICHPRNNDFYMCAHAGMIGTSRPTHYHVLLDDIGFSPDELQELV
HSLSYVYQRSTTAISVVAPICYAHLAATQIGQFMKFEDKSDTSSSHGGLTAAGVAPV
VPQLPKLQDSVSSSMFFC

>LotjaGi6g1v0002100.3
MPVRQMKEGSEQHLVIKPHLLNPMNSARKVTRAVQNGKGPPPPPQEPNNQTSPQ
ERAKGRRKSRVCRKSDQGGVLMRPCTVVTNTANGLVDNGSISGDIEMGYPSSSKS
LSFAPRPGFGQVGAKCIVKANHFFAELPDKDLNHYDVAITPEVSSKIVNRSIIAELVR
LYKESELGMRLPAYDGRKSLYTAGALPFSRREFKIKLIDVDDGVNPTKREREYCVVI
KFVARVNLHHLGQFLAGKRADAPQEALQTLDIVLRELSSKRFCPIGRSFFSPDIRTP
QRLGQGLESWCGFYQSIRPTQMGLSLNIDMASAAFIEPLPVVEFVGQLLGKDVLSR
QLSDADRIKVKKALRGVKVEVTHRGSFRRKYRVSGLTSQPTRELVFPVDGNSTMK
SVVDYFQEMYGFIIKYTHLPCLQVGSQKKANYLPMEACKIVEGQRYTKRLNEKQITA
LLKVTCQRPRDRENDILQTIQHNAYDQDPYAKEFGIKISEKLASVEARILPAPWLKYH
DSGKEKNCLPQVGQWNMMNKKMINGMAVSRWACINFSRSVQDNIARTFCNELGQ
MCQVSGMEFNPEPVIPIYNAKPEQVEKALKHVYHVSMNKTKGKELELLLAILPDNN
GSLYGDLKRICETELGLISQCCLTKHVFKITKQYLANVSLKINVKMGGRNTVLVDAVS
CRIPLVSDIPTIIFGADVTHPENGEDSSPSIAAVVASQDWPEVTKYAGLVCAQAHRQ
ELIQDLYKTWHDPVRGLVSGGMIRDLLISFRKATGQKPQRIIFYRDGVSEGQFYQVL
LYELDAIRKACASLEPNYQPPVTFVIVQKRHHTRLFANNHRDRSSTDKSGNILPGTV
VDSKICHPTEFDFYLCSHAGIQGTSRPAHYHVLWDENNFTADGIQSLTNNLCYTYA
RCTRSVSVVPPAYYAHLAAFRARFYAGPEELQENGSTGTGHGSKVTRAAGECGVK
PLPALKENVKRVMFYC

>Rhiir2_1|1764424
MEEVLFSQFVKRPSTCDLGAQIKVRANFFEVTRMQDTNISQYEVNITPTVPQRLNR
RVFNRLVEQYRERALGGARPVFDGSAIVFTHKPLPFETRSFDVELEEDNAAVGRAR
IPRRFTIRIRKTREIFMGDLFRFLNGRGDMTNNCQMAMVAMDIIISHKISAIYPTVRRS
FYTPQVTKPLPGGLEAWQGYFQSARPTRGRMMINIDLSTTAFYESGPLIQMVAKILR

LRSPNELRRGLSESDHLKVEDNIRGLRIMDNHRTGNRRKFKIEGLTEAPASHATFPR
DDGSRIDVRTYFQNQYNRRLSYPFLPCVIVRRNQYFPIEVCDVIREQRYMRKLTGM
QTDEMMNFARQNPNVRANKIQDGLNILNYRDNEYLQQFGMSISNNMAVVDARILPT
PTIQYHPTSRERRIEPKGGVWNLRDKKVATGATLGSWSVLAFLNERNLPDNIIEGFL
REFVVTCIDTGMNIPNRRPPICRENPIGNTEESLKKAWLRAGNNAKVQPQLILCILPN
TGQELYGEIKRISETVIGVATQCVQSVHMNRPKKQYCANVCLKVNVKLGGMNSFLI
PEHIPFITEKPTILIGADVSHPGPGETDKPSYAALCGSMDARASRYAASIRVQTGRY
EIIVDMANMVKELLKTFYQTCGRKPERILFYRDGISEGQFENVLRSELNAIRAACQAL
DAKYKPTITFIVVQKRHHTRLFPMDRQNTDRSGNCLPGTVVDKNITHPFEFDFYLLS
HAGLLGTSRPAHYHVLYDENGFDANRLQTLSYNLCYIFVRCTRAVSLVPPVYYAHL
VTTRAKHHTHKVQHTFGVVKPDLQRAMYFA*

>Rhiir2_1|1577331
MEEATFQITQFVLRPDIGDEGSNIRVRTNFFEVTNMQDTNISHYDVTITPTVPKRLN
WKVFNRFVEQYREEALGGARPVFDGRTNMFVHRQLQFESATFDVELEEEDVPVS
RTRPPRNFKIKIRQTRNIVMRDLFQFLQARGSLTSNCQMAIMAMDIIISHKISTIYPTV
NRSFYTPRITHPLSGGIEVCQGYYQSARPTRGRMMINVDLSATAFYESGPLVQIVA
NILDRTYNELYGGISENDRQKLEWIMEGLRIRDNHRSGNRRKFKVEGLTQTPASRT
MFDRGDGSIIDVRTYFQNAYNRPLVYPLLPCVIVRRNVYLPIEVCDVIPEQRYMRKL
NDEQIANMMNFTRQNPTIRANKIQEGLNILNYRRNEYLQQFGMSISTDMTVVNARIL
PTPTIEYHPTSREYRIQPKDGVWNLRDKKVATGATLGSWSVLAFENDTRLPDHVIY
TFVRELVSTCQDTGMNIPNRNPPILRANIQGTTEESLKLAWLRAGNTAKAQPQLILC
VLPNRGLQLYAEIKRVSDTVIGVATQCIQSIHMRNPKKQYCASVCLKLNVKLGGMNS
FLIPEHIHFVTERPTILIGADVSHPAPGDNQSPSFAALCASMDAKASRYAASIRVQAR
RYEIIADLANMVKELLKIFYQTCGRKPERILFYRDGVSEGQFNSVLQIEINAVRAACQ
ALEARYRPTITFVVVQKRHHARFFPMERQYTDRTGNCFPGTVVDVGITHPFEFDFY
LLSHPGLLGTSRPTHYHVLFDENGFNANSLQTLSYNLCYIYVRCTRAVSLVPPVYYA
HIVSNRAKFHLNSESGDDVTFGVVKQELQRVMYFA*

>Rhiir2_1|1516785
MEKETYQIIKSPGIGNKGEPIKIRANFFEVTKIPKMRITHYDITISPEVPPRLNRKVFER
FSKDNQDALGGVKPVYDGRFNMFTHKQLPFESKSFEVKLDKGSAPVSKTRPPEIFK
IAIKKARDIDMNDLFQFLNAKGKMTNNCKMAINAMDIIISHEVSAKYPTVRNSFYTSH
EAASLRGGIEAWQGYYQSARPTRGKMMINIDLSSTAFYEGGPLIQMVARILGLRSP
NDLRRGLSDKDHQKVERRIKNLRISDNHRPEYRRKFKILKLSQNSASDTTFDGDNG
NKIDVKTYFQNKYKKRLLYPFLPCVVVRNDNNLPIEVCDVIPEQRYMRKLDKDLTED
MMNFARQNPNVRANKIHAGLNILNYKNNAYLKQFGMEISNEMTVVNARILPTPTIQY
HQSSRENRVRPNGGSWNLRDKKVINGATLSSWSILSFLNSRFLPDQVIRLFVRELII
TCKDTGMTITEHEPPICRENPIGDTEGSLKKAWVMARDKARGVKPQLILCILPSNGI
DLYAKIKLVSDTIIGVATQCVQSTHARKPKKQYCANVCLKINVKLGGENSFLVPEHIQ
FLADEPTILMGADVTHPSPGDTESPSYAALCGSLNVKASRYAASLRVQRGGTEIITD
LENMVKELLKAFYQTCGKKPKKILFYRDGVSESQFMEVLDSELTAIKAACHSLEANY
KPTITFVVVQKRHHTRFFPIQGADRTGNCFPGTVVDRDITHPFEFDFYLLSHAGLLG
TSRPTHYHVLYDQNGFDANKLQMLSYNLCYVFARCTRAVSLVPPVYYAHLAAFRA
RLHSPRSTDTESSEASEGGRSAATTFGAVREDLRKVMYFV*

>Rhiir2_1|1582012
MASITEFVKRPGLGRLGRPVRVRANFFEVTALPDANIHHYDIDISPEVPPAMNRKIYK
HFEALHSEKDLGKIKPVYDGRKNLYTAKPLPFGESAAFDVTLPEDDGTPSGKRPAR
AFKVKIKLVNKINMEELHRFLEGKGSISPNILTGIMALDILIRHKPSMEHATFGRSFFT

KQGSRPLNGGVEVWQGYYQSARPTPKKMMINVDLSATAFYEGGSLVQMVVKMLN
KRSVDDLRRIHDRDRTKLEKSLKNLKVYVTHRGENASKRRLRITKLTNTPASNTKFE
VNGQQIDVATYFFNTYNKRLQYPFLPCVVVRKETYLPMEICNVVEGQRYMRKLNER
QTADMIKFTCQPPSARANKISQGLQILDYRQNEYMQQFGLKVSTEMAVVQARVLPA
PKLQYHPTSRDANFTPRDGSWNLRDKKVATGATLGSWACAVFGNERDYPMAAVQ
KFIRELVTTCQDTGMNIPNKNPPIQHCNPQGPIETSLRQVWVKAGNLAKSKPQLILCI
LPNTGVPLYAEIKRVSDTVIGVASQCIQGKHMFAAKKQYCANVCLKMNVKLGGMNS
FIDPTQVPFITQRPTILMGADVTHPAPGAENTGRPSIAAVTASMDAKASRYAASIRV
QTGRQEVISDLAEMVKELLKTFYQTCGRKPDRILFYRDGVSEGQFSIVLKDEVKAIK
EACKSLDEKYKPTITFVIVQKRHHTRFFPMESKDADRTGNCQPGTVVESVITHPFEF
DFYLQSHPGLQGTSRPTHYHVLLDENGFNSDSLQTLSYNLCYVFARCTRAVSLVPP
VYYAHLVCARARFHASGENWSDPDTSEGAGGVASYAAVKAELLKVMYFM*

>Rhiir2_1|1782262
MSDTQFLIGTFCRRPTLGNLGRQIRVRSNFFEVLALPTQNIIHYDVTITPDVPPALNR
KIFGEFERISREGPLNGIRPVFDGRKNIFAPRSFPFGDTATFNVTLPEDETIPSKRTP
RTFKIKIKKAGEIYMDELHRFLQGKHPMTSNCLTATMALDVLIRHLPSMRHVTVGRS
FFTNAGAEPLFGGAEVWQGYYQSARPAQEKMLINVDLSATAFYESGPLIQMVTKIL
GRRNPDDLRRGFSDKDRIKVEKTLKNLKIRVTHRGEATSKRNFKIIKLTPTPASQTM
FDAGNGVTTDVADYFARQYGRRLSYSHLPCVTVQKNIYLPLEVCEVVEGQRHIRKL
NEKQTADMIKFTCQSPHLRANKIKQGVDILNYRKNEYLKQFGLEVASEMIVIQARVL
PVPTLQYHPSSREHSFQPREGSWNLRDKKVAQGAVLGSWAIVAFGSERDLPTQSI
QAFIRELVITCQDNGMSIPNRTPPIMHTNSQGDIEGTLKTAWLHAGNAAKAQPQLIV
CVLPNTGVPLYAEIKRVTDTVIGVASQCVQSRHMMQAKKQYCANLCLKINVKLGGM
NSFISPSQIPFISDKPTILMGADISHPSPGEPSRPSIAALCASMDAKASRYSASIRVQT
GRTEIIADLANMTKELLKTFYQTCGRKPERILFYRDGVSEGQFDQVLNGEIKAVRAA
CMSLEESYKPSITFVVVQKRHHTRFFPLDKRDSDRTGNCLPGTVIESGITHPYEFDF
YLQSHAGLQGTSRPTHYHVLFDENTFTSDGLQALSYNLCYVYARCTRAVSLVPPVY
YAHIVCRRARFHARDQHWSDTESGSEGDQGTASIFGVVKPELQRVMYFM*

>Rhiir2_1|1884825
METISKTPSELTEFVLRPNLGTSGRHIRVRSNFFEITSLPDMEIIHYDVTITPDVPPILN
RKIFAEFEKLNLSGALGNVRCVFDGRKNIFASRQLPIKDSQVSLPNNQNTSGKPLPK
CFELKVKKVGKINYEELVLFLGGKRQLTPNCLTAIMALDVIIHHQPSMDYVTVGHSFF
TPRDSRPISGGAEIWQGYYQSARPTQGKMMINVDLSATAFYERGPLLLMITKLLGR
RTPDDLRRGVSDKDYQKIERSIKNLKIRVTHRGELVSKKNFKISKFTPSSADNTKFD
SNGVTMSVSEYFQKTYNRRLNYSFLPCVVVKRNIYLPIEICEVVEGQRHIRKLSEKQ
TADMIKFTCQQPHERANKIRQGLDILNYRTNEYLRQFGLTVNNDMVVLEARVLSTPT
ITYHPSSRDFAFQPREGSWNLRDKKVAVGATLGSWAVVCFGSERDSPPQMIQAFI
RQLVITCSDTGMDIPNKTPPIIHASPQGDIENILKQAWLRAGNISKAQPQLILCILPNIG
VPLYAEIKRVADTVIGVASQCCQNKHMIQAKKQYCANICLKINVKLGGMNSFISPSQ
VPFITDRPTILMGADVSHPGPGDLNLPSIAALCGSMDSRASRYSASIRVQSGRTDIIA
DLGNMAKELLKTFYQSCGRKPERILFYRDGVSEGQFSQVLRGEVDALRAACASLE
PGYRPTITFVIVQKRHHTIFFPLDKRDADRTGNCLPGTVIESTITHPFEFDFYLQSHP
GLQGTSRSTHYHVLYDENSFSSDGIQQLSNNLCYIYARCTRAVSIVPPVYYAHIVCR
RARFHSHGDDIEISEGGIGATSTFGVVKPELQRVRPVLENHLIRLQGLCARWRMNL
LAISDIDKNIMFVAMNDTIFVHRLNFNGKPSEPFKKLRYSTEPDYTNKSHTINAIKVGK
IGNEEVLVSVDEAGDIRIWFTSNLDKNPIQFSNNESTWGIALHGPKRLLAVSSNSHEI
TIFNLKESGKFFNEFDDDDDNLESSGEESQTPRTYASVLTKISESDALGEKAISVLR
GHEHNIPNICFSACGRFLVSCSIDSTCRVWNIKTGELMQKKILTGDWSDLNDNDEW

GWTANFVPKSAFKTVSSNCEELQHLISNPESIPSSPRSVETMDSLNLYRQNLRRNL
SGQRPNVRAPEDLYRIIISRNLRIRNREYEDGYIENGAHSESGTEEESTDDASELES
NEGDSELELSEDDTEVGSNNNDQHEYNEHSLNRERLTEIEGSATPAYYDRAESAT
PVVENYTENNPDYRWSVGHELNILNNNHEQNENNSSSIPERENHNEERSEEIIEER
SSQPTQSNTNTYESPAYPLYSPRSYTVSEDLTPVSSHNTSFSPELSGNLNNVSQSN
LPDSFTGSSLVYYHQNSQFSSFSPINPTHLPTSPTFSPQFSPQYVPTTSTQVSPFS
QHYSSTSPPTSPHYSPTSPYYTYLPTSPQYSSTFSTFPPSYNTSPQYATQYPSHNS
SNYSPSSPHFSPISPEHIDHPSWFSNIHHSYSPIVLQHSSSSVSPTYSPVSPEYIPAN
RELSPYHSYIEESIEDVINGLRQESSLSSPNSPNIRHDSSPERHEIEPISQSDHLDLH
DNDTICSHDSLTIDSPLPSSSRESSVNNIDSPLRTSNDYYIRSHVRIQPLGSSQSDCC
PDLGLPDELVLYTTKYDLLLLDPKLNLKILRTEKDLVHRVDTRRYPIHLDFDRLNMVE
VIPELSLMIVASQQGKVALTRLIRIVCDDGEENYYLHPEKYLPNTIMNTPLLGMFVAK
HNKLQDPALFYYRLYLMYINGTMFCYEIRRKKETNPLRMDNIYI*

>Rhiir2_1|1606291
MFNVTEFGKRPGFGRLGRPIRIRTNYFEITNFPDSNIHHYDIVISPEVPPTLNRKIFQI
ATENSFYGIKAIFDGRRNVYTIRPFPFGDAQALDVTIPEDNASRRIPRIFKMKIKKVNE
INMEEVYRFLDGRSSISSNILTGIMVLNVLIHHKPSREYLKVGRVFYTNQGSQSLSG
GVEVWQGYFQSIRPTPRKLMINIDLHATAFYESDSLVQLVVKILNKRSVEDLREIQER
DRVKLEKCLKNLKIYVTHRGESAPRRRFKITKLTNTPASSTMFEVNGQRIDVVTYFN
NTYGRSLQYPFLPCVIVRMNSYLPMEICNVVGGQRYMHKLNKRQIAEMIKFTRQPP
SSRADKINQGLQILDYRQNEYMQQFDFRVSTEMTITQARVLPAPTLHYHSASKEDT
FIPKDGLWNLRNKKVAIGATLSSWACAVFGSERDYPIGTIQNFIRELVNTCQDAGMN
IPNRNPPILHCNPQGGIENSLRQVWVRAGNSAKSKPQLILCILPNIGGILYAEIKRVCD
TLIGVATQCIQSRHIMRAIRIYCANVCLKINVKLGGENSFIDPSQIPFITQRPTIIMGAD
VTHPAPGEQNTPSIAAVTASMDAKASRYAASIRIRSGRQEVIGDLAEMVKELLKTFY
QTCGRKPERILFYRDGVSEGQFSIVLNEEIRAIKDACMSLDGRYKPTITFVVVQKRH
HTRFFPIDTGIDGDRTGNCPSGTVVDSTIVHPFEFDFYLQSHPSLQGISRPAHYHVL
LDENGFNADSLQTLTYNLCYNFARCTRAVSIVPPVHYASLVCRRARFYASGENWS
NPDTPEGVGSASYGVVKSELLKTMYFI*

>Rhiir2_1|1456683
MASITEFVKRPDFGRVGHPIRLRTNYFEVIAFPSSNIYHYDIVIIPEVPPTLNRKIFQVA
ENSFPGIRAVFDGRRNVYTIRPFPFGDAQALDVTMPEDNAGGRTPRTFKIMIRRVA
VINMEEINRFLNGRGSISSNILTGIMVLNILIHNKPSKEHIKVGRVFYTNQGSRSLSGG
VEVWQGYFQSIRPTPRKLMINVDLHATAFYESDSLVQLVAKKLNKKSVDDLRGIHN
RDRTKLEKCLKNLKIFDTHRGEVASRRRFRISKLTNTPASSTKFEVNGQQIDIATYFF
NTYNKRLQYPFLPCIVIRRETYLPMEVCNVVEGQHYMRKLNERQTENMIKFTCQPP
SSRANKISQCIEVLNYRLNEYMQQFGFRVSNEMAIIQARVLPTPTLHYHPASKEDTFI
PKDGLWNLRNKKFATGATLGSWSCAVFGSERYYPMGAIQKFIRELITTCQDTGMNI
PNKNPPIQHCNPQGGIETALRQVWVKAGNLAKSKPQLILCILPNTGVSLYAEIKRVG
DTVIGVASQCVQGKHMFTVKKQYCANVCLKINVKLGGMNSFINPSQMPFITQRPTIL
MGASVTHPAPGAENMGRPSIAAVTASMDAKAYRYAASIRVQTGRQEVISDLAEMV
KELLKTFYMTCGRKPERILFYRDGVSEGQFSIVLKDETKSIKEACRSLDEKYKPTITF
VVVQKRHHTRFFPTNARDGDRTGNCSSGTVVDSTIVHPFEFDFYLQSHPNLQGTS
RPMHYHVLLDENVFNADSLQTLTYNLCYNFARCTRAVSIVPPVYYAHLVCARARFH
ASGENWSDPDTPDTPEGAGVARYAAVKTELLKTMYFM*

>Rhiir2_1|1623940

MEEPFQLTEFVKRPGVGHVGKQMKVRTNFFEITSLPEMEILHYDVTITPEVPQQLN
RKVFDLFSEQNRTGALGNAKPVFDGRKNMFAHKSLPFGQAATFDVHLEEDDSPIG
RKRPPREFKIKIRKTGQGINMEELTQFLHARGRMTSNCQMAIMAMDVIIRHRPSTVH
TTVGRSFYTPQGAQLLYGGAEVWQGYYQSARPTRGRMLINIDLSATAFYEPGPLV
QIVAKMLGRRSPDDLQRGITDRERLRLERTIKGLKIKDTHRSGNRRKFKIEKLTPTPA
SQTMFDRGDGSNVDVKTYFQEAYNKRLLYPFLPCVIVRRNVYLPMEVCEIIPGQRHI
RKLGERQTADMIKFTCQQPSMRANKIRAGLDILNYRGNEYLQQFGMRISADMLLVN
ARVLPTPTIQYHPSSREHHVQPRDGSWNLRDKKVATGATLGSWAVLAFLSQDDLP
DLSVNTFVRELVNTCQDTGMNIPNRNPPVLHANPQGNIEESLKQAWLKAGNTAKA
QPQLIICILPNTGTPLYAEIKRVSDTVIGVATQCVQSRHTMQAKKQYCANVCLKINVK
LGGMNSFLSPSQIQFISDRPTILMGADVTHPSPGDRDRPSIAALCASMDAKASRYA
ASIRVQTGRTEIIADLANMVKELLKTFYQTCGRKPERILFYRDGVSEGQFSHVLKNEI
IAVRAACQALDNNYKPTITFVVVQKRHHTRFFPIDRHNADKTGNCPPGTVVESDITH
PFEFDFYLQSHAGLQGTSRPTHYHVLFDENGFSSDMLQTISYNLCYLYARCTRAVS
LVPPVYYAHLVCTRAKFHSRGEHWSDTESSEEGTGAAVTFGVVKQELQRVMYFM*

>Rhiir2_1|1868966
MEEAAFQITQFVRRPGTGRDGRNIRVRTNYFVINDIPQMNIIHYDVTITPEVPQRLNR
RVFDRFVEQHQGRALGNVRPVFDGRRNMFAPKLLPFGQAGTFDVDLEEDDAAAT
ARRRPPRTFAIKLRRVGQDIVMEELMQFLHARGTLTNNCQMAIMAMDVIIRHKPSS
DHITVGRSFFTPQGNRLIYGGAEVWQGYYQSARPTRGQMLINVDLSATAFYEGGP
LTQMVVKLLDRRTPNDLYRGMNDRERQRVERSIKNLRIRDNHRAGNRRKFKIEKLT
PTQASRTMFDLGDGGQTDVVTYFQRQYNKRLLYPNLPCVIVRKNVYLPIEVCDVIP
GQRHMRKLDERQTADMIKFTCQPPNARANKIQAGLEILDFRNNEFLREFGMRISND
MMVVNARVLPIPTIKYHPTSKEHDVRPNGGAWSLKDKKLATGATLGSWSVLAFLNQ
NELPDQAINPFVREFVNTCQDSGMNIPNRTPPILHANPIGNIEEALKEAWLRAGNTA
KAQPQLIVCILPNKGTPLYAEIKRVSDTVIGVATQCVQSRHVQQAKKQYCANVCLKV
NVKLGGMNSFLDPAQIPFITDRPTILIGADVTHHGPGEDDKPSIAALCGSMDAKASR
YAATIRVQTGRFEIIADLANMVKDLLKTFYQTCGRKPERVLFYRDGVSEGQFKHVLQ
SEINAVRAACQALEASYRPTITFVVVQKRHHTRFFPLDKRDSDRSGNCLPGTVVET
GITHPFEFDFYLQSHAGIQGTSRPTHYHVLFDENGFNADSLQTLSYNLCHIYARCTR
TVSLVPPVYYAHLVTTRAKFHTRWHESESSEGGAASAFGVVKQELQKVMYFM
*

>Rhiir2_1|1641401
MASGATKAAANIGMIVAKRPEKPGSAGKVVELKTNFIAVSTEFKYPKVHYYSFEVSD
AKKKKAGRKDSQKVFEQMKKNQKFGAKALPVFDGGQVYSISQLDIGHRKETRSHS
VKIPVDADGSGREKDLKVEVKYQGEIDLSPIGDYVKEKSTSAWDGNIQSSLTVLNAF
ANNKVRLSFLSVGRKAIFPPPRDNRPIFLPGGIEMKQGFYQSVRPGWGRLTINVDIC
ATTFYPAGPIIDLIPKIIKTKFDRHEKTRDELRRGLDRREIDTLNRFFRGVMIFVTHRS
DNRKPKHKVSYLDSQPADQAKFKQGNKEITIAQYFKETYKQTLQFGRLPCIVVIKPD
KTKVFFPLEVCEICPGQRYEDKLSDDARREMVQKTAIKPWERFDRIETAIKDIFRHQ
RDENLRSIGMNVDPEFIKVKGRVIQPPRLTNGTEIVPDGGRWDLRKFIKCPRLHNW
SVIVFENRNYAPLEMVKNAIRQLVSSLSERGMDVYPDPPIEYGNPQGGYDKSLLLA
AQKARITRDRPAQLIICVIQKKVLGASGIHAIIKRICGTQLGVISQCIVANNMKGQNPS
RWRQICGNISLKINGKLGGKNCVLADRELNFGTNKPYMVFGADVFHPGREEKGRP
SVAAVCASVDKTVTRYVGRHSMNQKIKNEIIENLEGMAVELLNQFQRENNILPFQIV
FYRDGVAEGQFQHVVQKEVEALKRAFSRVYGEKGEKGQKPQLTFIIVQKRHHARF
MPVQRGDSDRSGNCRPGTIVDTEIVVPQEFDFYLQSHASPLGTARPTHYHVLLNEA

NFPVDAIQTLTYKLCHLSARCNLAISHVTPVHYAHHIANQAKHFVSWDGSGSGRSG
SSGGRGGDTVPNTTRCEKMKSGIENEMFFV*

>Rhiir2_1|1741331
MGLNVTDFVKRPSLGREGREIRVRANFFEITSLPEANIHHYDVTITPQVPPLLNRKVY
QVFEKSNCNNKAVFDGRKNIFTCERLSFEDSATFDITLPEDDGSTSTRRQPRVFKV
KIKKVNEINMEELQRFLDGKAQCSSNVLTAIMALDVLIRHKPSMQYVTVSRSFYTRE
GSRNLSGGVEAWQGYYQSARPTPGKMMINIDLCATAFYESGPLVNVVVKMLGKQ
HPDDLRRGINERERAKLEKELKNLKIRVTHRGEKNAKRRYRISKITPQDAQRTKFDD
ADGHKMDVASYFQKNYKRLTYPHLPCIVVKTDNFLPLEVCEVIEGQRHFRKLNEKQ
TADMIKFTCQQPNIRANKIKMGFDTLNYRHNEQMNKFNMEVLNEMATVRARVLPTP
TINYHPSSKDATFIPRDGVWNLRYKKVVTGATLGSWSVLVFGSEREYSAHSVQNFV
RELVTTCSDTGMNISNKNPPILHANPQGNVEESLKQAWIRAGNAAKSKPQLILCILP
NTGIQLYGSIKCAGDTVIGVVTQCIQGRHMLKAGKQYCANVCLKINVKLGGTNSFLA
PNQNPFLHEKPSILMGADVTHPPHGSNRPSIAALCASMDSRASRYAASIRQQASRT
EIISDLADMVKDMLKTFYQTSGKKPERILFYRDGVSEGQFEQVLNEEIKAVKSACHS
LDPKYSPTITFIIVQKRHHARFFPIDKKDSDRTGNCYPGTLVETDIVHPVEFDFYLQS
QAGLQGTCRPTHYHVLYDENEFTPDSLQTLSYNLCYIYARCTRVVSLVPPVYYAHL
VCSRARFHIRGDNFSEESSTEGDEVAFGAVKPELLKVMYFM*

>Rhiir2_1|1489847
MYTNPQGEITMALNIACHKSLFNKNYHPHIIICILGSTGPIYGEIKRIGDTQLGVPTQL
NAKLGGQNVILTNDQIDFVSSEPTVIFGADIFHSGRGDNRPSIAAVCVSLDSKATRY
GRFSVINKDPRNEIIEDLKGIVIDLLRVFYQRN*

>Rhiir2_1|1779081
MEKETFQIIKRPGIGNEGEPIKVRANFFEVTKMQKTKIIHYDVTIKPEVPPRLNRKVFN
LFSEKNQEALGGVRPVFDGRTNMFTHKPLPFEGKSFDVKLEEGNVPVARKRPPED
FRITIRKARDIDMEDLFQFLFAKSRMTENCQMAITAMDIIISHEISAKYPTVRNSFYTP
QGARSLFGGIEAWQGYYQSARPARGKMMINIDLSATAFYEEGPLIRMVAKILDLRSP
NDLRKGLSDKDRQKVEKRIKNLKIRDNHNPENKRKFKIEKLTKTPASHTTFDKDGSR
IDVKTYFQNQFNKRLIYPFLPCVVVRKTVYLPLEVCDVIPQQRYMRKLDKNLTDEMM
KFTRQNPNVRANKIHDGLNILNYKGNEYLKQFGMEVSKDMAVVNARILPTPTIQYH
QSSREHRVRPNGGAWNLRDKKVANGATLSSWSVMVFSNYKHLPDQAIKFFVREL
VNTCQDTGMNILDRDPPICRGNPIGNIEESLNKAYLNAKEKARVIKPQLILCILPNAGP
ELYAEIKRISETAIGVATQCIQSIHMKNPKKQYCANVCLKMNVKLGGENSFLIPEHIQ
FLIDDPTILMGADVTHPTATADDESPSYAALCGSMNVKASRYAASLRVQTGGTEIIID
LENMVKESLKAFYQTCGLKPKKILFYRDGVSESQFMEVLECELTAIRAACRSLEASY
KPNITFVVVQKRHHTRLFPIHGGDRTGNCIPGTVVDMDITHPFEFDFYLLSHAGLLG
TSRPAHYRVLYDENGFDANRLQMLSYNLCYVYARCTRAVSLVPPVYYAHLAAARA
KIHSPYRDTDTESSKGDKSTATTIGEVKGELRKVMYFM*

>Rhiir2_1|1829955
MEKESLGEPVKIRTNYFEVTKMPKMKIIHYVIMIKPEVPPRLNRKIFNHFSEKNQEAL
GGVKLEKGSEPISRIRSSETFKITIRKARDIDMEDLSQFLHAKGKMTNNCQIAITAMD
TIISYEISAKYPTVCKSFYTSQGARSLFGDIEAWQGYYQSARPTMGKMMINIDVIATA
FYESGPLIQMIAKILGLRSPKDLREGLSDKDRQNVEKRIKNLKISDNHRVENRRKFKI
MKLTQTPASYTMFDGVDGSKTDVKTYFQNKYNIRLLYPFLPCVVVSKNKYLPIEVCD
VIPGQRYMGKDTNLANEMTNFARQNPNIRADKIKACLNILNYKENEYLKQFGMEISN
DMTVVNARILPTPTIQYHQSSRENRVQPNGGSWNLRNKKVINGATLGSWSVLVFS

NSSDQAIEFFVRELIVTCQDTGMNIFERKPPICRENPIGNTEESLKKAWLRAGNSAK
AKPQLILCILPNTGSHLYAEIKRVSDTVIGVATQCVQSTHTIKPKKQYCANVCLKMNV
KLGGENSFLIPEHIQFLADEPTILMGADVTYPLPGDIKCLSYAALCGSMDAKASRYA
ASIRVQSGRTGIIKDLANMVKELLKIFYQTCGRKPKKILFYRNGVSKSQFIKVLECELT
AIKDACQKLEANYKPTITTGNCFPGTVVDMNITHPHPLEFDFYLLSHAGLHGTSRPT
HYYVLYDQNGFDANKLQMLSYNLCHTSVRCTCAVSLVPPVYYAHLAEISFGVVKKE
LRKVMYFI*

>Rhiir2_1|1755567
MSNKKIVQTFQIIIRKVGKDIVMEELVRFLNEKSSMTNNCKTAIMALDVIIGYQVSTAN
VTVGRSFFTQKESSPIAGGVEVWRGYYQSARPTRGKMMINIDLSATVFYQDGPLIQ
LITKILKLGSLDKLRSGLSERDHQMVERTIKNLKIRVNHRAETRTSSFKIEKLTQTPAS
STMFEKDGNTINVGTYFQNTYNKRLIYPFLPCVVVRRHILLPLEVCDVIPGQRYMRK
LNGGQMDEMMALTRQNPSIRTNMIRKGVKILNDQTEFMQQFGVKVSDKMAVVDAR
VLPAPTIIYHPSSREKHITPINGNWNLRNKKLAIGATLGSWCVLAFLSSDVFPDNVIST
FVRELATVCQDTGMNVPNKNPPILHANPQGNIEESLKQAWLKAGNSAKAQPQLILCI
LPNTGQPLYAEIKRVSDTVIGVATQCVQIKHTRKTKKQYCANVCLKINVKLGGMNSS
LSPEQISFISDRPTILMGADVTHPAPGSGNERPSIAAVTASVDSKASRYAASIRIQRG
RTEIIADLGNMVKELLKAFYQTCGRKPERILFYRDGVSESQFENVKAIEVEAIKTACH
ALDNKYNPTITFIVVQKRHHTRIFPIDGGDTDASGNCLPGTVINTVITHPNEFDFYLQ
SHAGLLGTSRPAHYRVLYDENGFNQDALQTLSYNLCYLYARCTRSISLVPPVYYAH
LVTKRAKLHAR
GGSFSDTESEESAGGAAVAFGEVKPVLQRVNITNK*

>Rhiir2_1|1662120
MSNERVRRRVNEKVKEKLKEIETQPEIRDPNEPPVPILTRKEYGDGYGKVGRHVEV
KVNSFEVTNISKYNKFFKYNIEINPIDKDIEVDVKRERSDYGKSIRRRVLKELSLEMED
WFHKVVYAYDGGSTLYTTSYLGFDKVKDVKAVVKEEISIANLQNLDGEPTRYKVIIN
RLDTIIELTQINKYINGEEFEWEFFSDEAFNVLNSLIHKVGMENEKYIQSGNSSIYNKE
NKKNINGGVELCLGWFSTVRPGQGSLFINVNPTYTTFYQPLNLEEFLFQCFFPKYQ
NIPNEINAFQLKKINEILKGLLVKPLHLEPIQTGRRIRRLLAATKVNRFRYKPRADSEE
TFPLERYFRERYKLKASSRLFVELMTDDDTSIAWPVEFCKIIEGQRYSNKKLSGKQR
GEIILLAKFKPSINEQETIRGAKEILQLQNDPTGVGMKVDPNLAKVDARVIKSTDVKY
AGKIMKPNNGQWNLKDVKFVESGERLEFWHVVIFEQSKWMPKGEVINFIENLARQ
CESQGMSIARNRPEIQYLQYYNKGPNVVKQAYEDARDILRRPPQLIIFIIPGEKSPINT
ELYETIKRVMDTEIGCLSQCVSVDSRKNFRSPQYCANLAMKINLKLGGVNSILPEDK
LRLPSGEEVLFLGADVTHPKDKNGKSICAVVGSLDNQAGRFITKYQDQERSGKEEIL
EIDKLIKEILREYCDYQKRRSRVKNNEVITLPPCIIMYRDGVSESQFERVLRWELPKIR
EACRLFRQGYEPKIIFSVVGKRHHTRIFPVDPKNRMEADKNGNCLVGTIVDKKITHP
TLNDFYLQSHFANQGTARPSHYTILHDDIKLTIDEFQGLSNTLCYNFQRSTSSVSIPA
PTYYAHLTCARAKMYLTTHRGNIYLPNVHRNLKNYPMYFM*

>Rhiir2_1|1848686
MYTNPQGEITTVLNIACHKSLFNKNYPSHIIICILGLTGPIYGEIKRIGDMQLGVPTQCI
LLKRLVRKNGIDQICSNIFLKVNAKLEQTVIFGAEVFHSGRGDNRPSIAAVCVSLDSK
ATRYGRFSVNKDPRNEIIEDLKGIRSWLQLGNQNPKYSFKKRNSKL*

>Rhiir2_1|1641155
MTTHLEKQLEKITVKEGVAPRPGIGNLGQKVNLRANHLKISKFPNLKLYLYNYNVTS
SSGRTIGKKVRWKVFEEIKKQKKFGKSLPIFNWNDMIYSSTKLFLEQDEFELPLPPS

EPAGKITTFKIKIKFKEEFSLQHIKDFMESKQAWNKNVQTCMNALNAYLNYKVRTNY
VSLGRGIYASNNNRVILSGGAELRQGHCQSLRVGWNDLTVNVDVCSGIFCPPGNV
VDIAAGILNRRKDDLRRGIDDRDRRMLAKALKTLRIRVLHRGDNKRSIYSIDDLSKDS
ADNIKFRDEEERETSVTQFFAKKYGMRLAFGSLPCIKVSKNCFPLEVCEILPDQPFK
GDITDNGRADMIKFTCVKPRERFQSIRDAIHNVFRYGQDENLRSINMGVDTEMIVVE
GRVLPPVSISFNKDRGQQNQKVDTGRWNFVSQVIQDGKKLTNWSILALSGDHQNA
IAQFGRQLTETLNKKGMNIVNQPAVVIFNQHGDIKRGLTQAAERARMNKDEPVQLV
LVVMRERSRLYSDIKRIAETELSIRTQCVLNKNIRKPKGFEQFCVNVGLKINAKLGGR
NYSLSTGQIDFVSSVPTMIFGADVYHSGVHEQHMPSIASVCASMDAAATIYSGRYS
MNKEPRNETIEELDLMVIDLLKAFRAKNGRLPQRILFYRDGVSEGQFRKVMEVEVD
MLRKAFKGGYGDNPPKLTFIIVQKRHHTRFEPTNQRDSDRGNCRPGTVVDTGIVVK
QEFDFFLQSHASLLGTGRPTHYRVLVDDNKFKADDFQSLTNKLCYLNARCTSSISIV
TPAYYAHLICNRARHYTVWQGESSSEGSATCASVSKPLTNLMYFL*

>Rhiir2_1|1697341
MAKNGRKEVEQRKGEEKKEPQIRDEQQEALREKAIKNMAKRPDYGKTGRHTHVLT
NYFEVKKIANKPYLEKYDISMKLIIQQRPEGEGNQRRRAPKQPKQQKQLPVHVQRM
VFQQLEKQERNGWFKGIGVVFDGNTTLYSTDLLRLNSPDSGNTSVSLKDDQDSRG
NPMEYKVEVQKLDRVDLGELKKCLEGKEMKWEYFDLVGIRGLNALIHHIPSMKYTQ
FGESTYLPSTRKSLGGGVELWMGWFESVRPGQDSYFVNVNTTYTVFYEPGILSNLI
PKYLNSSIPERFSQNQHDRVTELIRGLQFRPVHRPQVNTRLKIKRLCPNNAYDIKID
MPDSGEKVDIYTYFQHRYNITLRHVHLVELEGRRNDKVPIELCNVIEGQRFPVAKLT
SAQRGAMIKHTALRPQENVHRINEGVQDVLQFEKDFKLSSFGMEVDEQMAIIPARV
LKPPQISYHSTSKAGGTVRPKEGGWNLRDRKFVRSGNTLRYWVVVAFIDQRKFNQ
AKQFVKELVNTSRQQGMDIAETNPRINCVKPHGDAQTYQRMIEAEYNNARNHLLN
DLQLMLFVLPDDDEKRYRAIKYTTDTIIGVPSQCVQLDKVYKTSKQYCANVALKINLK
LGGTNQSLEETEIPQLTKEPVMLLGADVTHPTGGRAGPSNMPSICAVVGSLDRQG
GRFISKLESQRTRQEKIENMGGMVKEILIDYRSKNNILPLRIIMYRDGVSESQFEMVL
THELEKIKDACKEIDETYNPPITFVIVGKRHHTRFYPQQKNDTDSKGNCVAGTVVDR
KITHPYLFDFFLQSHTSLHGTSRPAHYHVLFDENKFTSDILQNLTHKLCYNYQRATR
SVSIAPAAYYAHLAAKRARLHLEQQPRGETEFVLKEVKESLAKDNTMYFI*

>Rhiir2_1|1745457
MEEAAFQITQYVRRPGTGREGRVVRVRTNFFEVITMPETNISHYDVTITPKVPQRLN
RKIFNRFVEENQRELRNTRPVFDGQSNMFAFRKLQSDASTFDVELAEENVPVGRT
RPPRRFTVRIRKTREIILEELFQFLNARGSMTENCNMAIMAMDILISHKIAAIYPTVRR
SFYTNQGARPLSGGLEAWQGYYQSARPTRGKMLINVDLSVTAFYESGPLTQMIAKI
LGFRSTDDLRRGFSEGDHHKVEDFIRNLRIRDNHRTGNRRKFKIEGLTRTPVSNTM
FDRGDGSNIDVGSYFQNQYNRRLLYPFLPCVVVRRNQYLPIEVCDVIPEQRYMRKL
NRRQTDDMMNFARQNPNTRANKIQDGLNILNYRDNEFLQDFGMRISNEMAVVEAR
VLPTPTIQYHPTSRENRVRPRDGAWNLRDKKVAAGATLGSWSVLAFLNERELPDNI
IEGFLREFAVTCQDTGMNIPNRRPPICRENPIGNTEESLKKAWLRAGNNAKAQPQLI
LCILPNTGQELYAAIKRIGETVIGVATQCVQSVHMGRPKKQYCANVCLKVNVKLGG
MNSFLIPEHIPFITEKPTILIGADVSHPPPGETDKPSFAALCGSMDARASRYAATIRA
QTGRFEIIADLANMVKELLKAFYQTCGRKPGRILFYRDGVSESQFRHVLQNEINAVR
AACQALEPNYRPPITFVVVQKRHHARFFPMDRQNTDRSGNCLPGTVVDVGITHPF
EFDFYLQSHAGLLGTSRPAHYHVLFDENGFNADSLQTLSYNLCYVYARCTRSVSLV
PPVYYAHLVTNRAKLHMLSEPGGATTFGVVKQELQRVMYFA*

>Rhiir2_1|1791259

MGHWTVYESILNSEYTITKMKLKENQGENLDKLLRNMGISHKMSKEYFPAMDVEVA
NRLAEMINSEGPKYKFDIPLYDGWAKFYGYKLPTFSASDAVYGLITLLKTKPSASIEF
GVEIQWVNDFNGRFEWLNNFHTALDALDRFFQINKMLKRRIHYDYETIISNSDSKDE
IELPKKQKAVDFKLTSTELKVAERPDVGLRGKSVDLKCNYLKINTLPNVDIHHYKFSV
RPCIKKPKALFKIFSRMCLDNVFGPVHPVFDGKEAIYSPSHLNINAINEDGEIYVALP
RDEKHEEIRTYNARIEKVQIINFRLLQDYINGQSPLTGEILNCNHVLNIIMNFTAHNMFI
VDMASEDWSDHMAKNRVPGANQGFCQSMRPGWENMVINVDFHDPNLKIPAESLI
NVVAKILNKTDEELLSKTLSEDEIKKLERTLTGLQIRIVHRGNPKTKYIIDGLSKELTKD
IKFRDDKGFLVKAVDFFPREFQWPLRYTLLPCLIVKKKLFMPMDVCEVMPGQKWEF
HPEDDSMLGMIKISTENQARFQHVESRVKNILKFFNTENIKELGMDIDNRMMTVNG
RVLNPPIITCDEGGQQTEVQTEKGRWTFENQVVKIGKPLENWSLVILCGERHNRFD
SIQEFLNQLCNMLNEIGLNVITVPEVMYANKQGNIEQALAIAYQKAHINKKISPQLIVCI
MPTHSKQLYSEIKRVSDTVLGIPTQCITADKVTFKWNKQLLANIGLKINAKLGGHNW
SLSKSDLSLITEVPTMLLGADISHPNVLTKVKRSITGMCGSMDLSATTYIGRSSVQKE
THNPTIEVLEELVSELLFNYKERNKVLPQRILYYRAGLTPSQFRGTLNAEIISLKAAFT
KTYEKNMPKLTFIIAEKSNHTRFLPDNPQDADRFGNCQPGTVVDKGIVFEKEFDFYL
QSHPGINSKARPTHYHVLYDENNFNSDLIQSLTYHLCYLSATCTHAISFVPPIAYARN
IAERARMYATAANTDLISQFEAMDIDDEIETKDNENQSFIEIENGSNKEPDYLFPSVS
RSLLEWRRDELLGIRTLPASNSTLAERAICPGGFIDCLNGGCCPRGSKCIADNKCSI
RCTPGAPLCNGGCCLFGQVCGGKFCVAGTKPKAPKPPPPAEKKAPPPPPKEKKAP
PPPPKEEKAPPPPPKEVKAPSPLPKEPQPKKQDLPSAPPPPPLPSFSPAPPPPSSP
PSPPKSPSSTKAPIKTPNSSVAGSLASSGSTFGSAFGSTLVYSTVLVYLNLI*

>Rhiir2_1|1748319
MPENNIIHYDVSITPEVPPRLNMKIFESFVNQYRERALGNARPVFDGRKNMLAHKLL
PFGDAATFNVTLEERTPVASRRPPREFRIRIRRVGDIVMGELFQFLRARSRMTNNC
QTAIMALDAIIGHKVSTLHVAVGRSFFTPEGSRSISGGAEVWQGYYQSARPTSDKM
MINIDLSATAFYEGGRLVQIVAKILELRSPDDLRRGLSEREHQKVERKIRNLKIRVNH
RAEARTSSKIERLTQTPASRTMFEKDGNTIDVATYFQTTYNMRLIYPFLPCVIVRRNV
FLPLEVCDVIPGQRHMRKLDGKQVDEMMNFTRQNPTLRANKIRAGLNILDYRNDEY
MQQFGVRVSNEMAMVDGRILPTPTIHYHPSSRDNRIQPAGGVWNLRDKKVATGAT
LGSWCVLAFLSPDVLPDHDINAFVRELANTCQDTGMNIPNKNPPILHANPQGNIEES
LKQAWLRAGNSAKAQPQLILCILPNTGQPLYAEIKRVSDTVIGVATQCVQNRYIRQT
KKQYCANVCLKINVKLGGMNSFIDPTQIPFISDRPTILMGADVTHPAPGPGSDERPSI
AAVTASVDAKASRYAASIRIQRGRTEIIADLANMVKELLKAFYQTCGRKPERILFYRD
GISESQFGSIITTEIAAIKAACQALDANYKPTITFVVVQKRHHTRFFPIDSRDADRTGN
CPPGTVIETVITHPFEFDFYLQSHAGLLGTSRPAHYYVLYDDNGFNPDMLQTLSYNL
CYIYARCTRSVSLVPPVYYAHLVTNRAKLHARGGPFSDTGSEEGGGAAVTFGVVK
PELQRVMYFA*

>Rhiir2_1|1668119
MAQRPGYGKEGRPIKLKTNYLKVVQFPNISLYHYSFDVAPKINKQTINKIYYEVANKN
NFGKFFAAFDGNSSIYSSTPLPLANERKGNKFKVVIGSNGDKTIKKGSYEATVKFIET
LSLDRLRNYISSQGPITSMVITCLAALNAYINFNVRQKYLSSGKGIYPPSPKNIPIILSS
GVELKQGFYQSLRPGCDQLFINIDICAAVFYPSGSLPEVIGKILSKRSTEELRFRGLS
NSEIILLSKYLKNLNVETLHHHNRKPVYKIKRITKESADNLKFYNDEKNSDMSISEYFL
TKYEITLKFPNLPCILVNRGIYLPIEVCYVLPGQRFEGPLPDITLSDMIKHTCVKPQER
FQRILNAVSDVFQHDRDPHLESIGMEVDCKNMVVLNGRVLNPPRLTFNEKNRQSE
FIPENGRWNIVNKVVCQGAELVNWAVVVFDQERSAPQQLVKSFMKRFREIANQKG
MNIPNDPYVMYANPQGEITTALNIACHKSLFNKNYPPHIIICILGATGPIYGEIKRIGDT

QLGVPTQCILLKRLTRKNGIDQICSNIFLKVNAKLGGQNVILTNDQIGFVSSEPTMIFG
ADVFHSGRGDNKPSIAAVCASLDSKATKYAGRFSVNRDPRNEIIEDLKGIVIDLLRVF
YQRNQVLPRKILFYRDGVGENQFQHVKTYEVKVLKEVFASVYRNSGPTLTFIILQKR
HHTRFMPTEPREGDKLGNCRPGTVVDRTILVEQEFDFFLQSHSSLQGTSRPIHYNV
LHDENDFTADGIQTLTYRLCYLSARCTLSISQVPAVYYAHLIANRAKHYFRWDGDEK
SDLSRGSGGSSVGSIEEVKNGLTNAMFFI*

>Rhiir2_1|1851280
MEKETFQIMKRPIERGPIKVRTNFFEVTKLPKTKITHYDVKISPEVPRRLNREVFNRF
SEENQEALGGVKPVFNGRADMFTHKQLPFESKSFEVKIEQVNALVTKKRLPEIFKIAI
RKIGVIDIEDLFQFLHGKGNLTNNCQMAIYAVNIIISQEISANHPTLLYNFYTPQGATSL
HEGIEAWQGYHQSAHPTRGKMMINIDLHAAAFYEGGPLIQMVAKILGLRSPNNLRR
RLSDMEHQKVEKRIKNLRISDNHRPENKQKFMIEKLTQNSASDIMLEVIGNKIDLKTY
FQNIYNIRLLYPFLPCVVVRKNIYLPIEVCDVIPEQRYWRKLDCNQTNKMTKFARQN
PTIRANRIQTGLNILNYRDNRYLKQFGMEVSSDMTVVNARILPTPTIQYHQSSRKSH
VRPNGGLWNLRDKKVINGAILSSWSILAFLSYKDLPDQAIKLFIRELITTCQDTGMNIS
ERDPPICHANPIGNTGESLKKAWLKAREKAWGVKPQLILCILPNTGSNLYAEIKRVS
DTIIGVATQCVQSTHTKKPVKQYCVNICLKMNVKLGGENSFLIPEHIQFLADEPTILM
GADINYPSSGYNESPYYAALCGSVNVKASHYAASISVQPIFSEIIVDLANMVKELLKT
FYQTCGLKPKKILFYRNDVSKIQFKYVLERELTAIKEACQSLEADYKPTITFVVVQKR
HRTRFFPIERKNADRSGNCLPGTVVEMDITHPFEFDFYLLSHAGLQGTSRPTYYQV
LYDENGFDANKLQTLSYNLCHIYARCTRAVSLVPPVYYAHLAGKRARLYSFHYTDT
ESSEGGKNVAVAVREELRKVMYFI*

>Rhiir2_1|1556957
MSKETFQIAKRPGFGNKGVPIRVRTNFFEVTKIPDMKIIHYYVTIFPKVSLRLNRKVF
NHFSEENQRVLGGVKPVFDGTRNMFTHKSLPFQDSSFDVKYVELEEDNAPMGSK
RPFRRFTIRIRKTRDIFMDDLFRFLHAKGNMTWNCKRAITAMGTIISHEISINHPTIRN
LFYTSQGARPLFGGIEAWPGYYQLVRPTRGKMMINIDSSATTFYEGGPLIQMIAKIL
RLRSPDDLRRGLSERDHQKIEKIIKNLRISDNHIPENRRKFKIEKLTQSSASNTMFNR
NKINVTTYFQKEYNRRLLYPFLPCVVVGKNYYLPIEVCDVIPGQRYIQKLNEIQTAEM
YKFTCQPPSTRANKIQAGLNILDYRNNEYLKQFGMAVSNNMTVVNARILPTPTIQYH
PTSRENRIEPKHGVWDLKNKRVATGATLGSWSVLAFSNERELPNQAIKHFLRELITT
CNDMGMNIVMRDPPICHVDPSENTEESLKKAWFMAKEKARVKPQLILCILRYKSAL
YAEIKRVSDTVIGIATQCILSNNIFRGKRQYYANVCLKMNVKLGGENSFLIPEHIKFVA
DQPTILMGADITNSSLGDYKSLSYAALCGSMNARASHYIASLRVQTGHAEIIVDLEN
MVKELLRTFYQTHRLKPKKILFYRDGVSKSRYIDVLESELTAIKDACHSLEANYKPTI
TFVLVKKRRHTRFFPIDRQNYDRTGNCPPGTVIETDITHPFEFDFYLLSHAGIMGTS
RPTHYQVLYDENEFDANRLQTLTYNLCHTYARCTRSVSLVPPVYYANLAATRAKLY
SSYDSDTGKTTFEAVKEALRKVMYFI*

>Rhiir2_1|1600861
MKKETFQIMKRPGIENEPIKVQTNFFEVTKLPKMKITHYDVTISPEVPPRLNRKVFAC
FSEENKNALGGVKPVYDGKANMFTHKKLPFESKIFEVELEKGSAPMTKTRPPQGFK
IVIRKARDIDMEDLFQFLNAKGNMTNNCKMAINAMNIIIRHEISAKYPTLRNPFYTPQG
ATSLREGMEAWQGYYQSARPTMGKMMINIDLSATTFYEGGPLIQMVAKILGLRSPN
DLRRGLSDMERQKVEKGIKNLRINDNHRPENKRKFKIEKLTQTSASDTMLDVDNSN
KINVETYFQNKYRRLLYPFLPCIIARKNIHLPIEICDVIPEQRYMRKLDRDLSDKMTNF
ARQNPTIRANRIQTGLNILNYQNNEYLKQFGIEVSNNMAVVNARILPTPTIQYHQSSR
ESCVRPNCGSWNLRDKKVINGAILSSWSVLAFLSYKDLPDQAIKLFVRELITTCQDT

GMNISERDPPICRANPIGDTKESLQKAWLKARDKAWGAKPQLILCILPNTGLYLYAEI
KRISDTVIGIATQCIQSKHTNKPVKQYCANVCLKMNIKLGGENSFLIPEHIQFLVDEPT
ILMGASVTHPLSGNNEGPSYAALCSSVNVKASCYAASIRVQSHTDILTDLANMVKES
LKTFYQACGLKPKKILFYRNGVSKSQFMYVLENFIPAFFLTYLILQTRVKKNADKSGN
CLPGTVVDMDITHPFEFDFYLLSHAGLHGTSRPTYYQVLYDENGFDANRLQTLSYN
LCHTYVRCTRAVSLVRF*

>Rhiir2_1|1662010
MSQTKINIKNESSVPILKRDGFGTKGRVVTVKANSFEVTKISKYDEFLKYTLEIKPIDA
EKNIEADIKRERSDYGKSIRRRILKELSLKKGEWFQNVVIAYDGGTTLFTTDRLGFDK
ETETKPVVIEIIILKDLHNINGKSTQYSVQISRTDTVVKLTQLEKYISGEEFEWDFFSD
DSLDVLNALIHQRAGEKFIKSGKSSIYNKGNKTKITGGIELWSGWFSTVRPGQESLF
VNVNPTFTTFYQPLRLDEFLLQYLYPKYQNLPDEFSDYQLKDINEILKGLLVCPLHLE
SVHTERRIKKLLPATKVAHFEYKPMTDSEETFPLEKYFEDKYQHRETKKLFVEIMSE
EGTHVAWPIEFCQITEGQRYKTNKLTGKQRAEIISAAKMKPYEHEKETIKGAHEILQL
HKNPTGVGMEVASHLAEVDAHVLESTAVEYAENVTIPANNGKWNLKNVKFVKSGE
MLAFWQVVVFEQQEKRISEEDVYSFIEKLITQCKIQGMNITKNIPHIQYVQYHKDDLD
KVIKSTYEDAKKTLNRPPQMMLFIIPGDKIQSTNTKLYETIKRVMDTEIGCLTQCVSV
NSNYKKLFNNLQYCANLTMKMNLKLGGVNSVLPEKKIGLPSGEDVLLLGADVTHPK
DKNGCSICSVVGSLDNRATRFITKYQVQERSGREEIINIDKMIKEVLEDYCNYKKNKK
GNVKNTVASLLPPCIIMYRDGVSESQFERVLKWELLKIKEACAEFQAGYQPKITFAV
VGKRHHTRLFPGNPKEADKNGNCLVGTVVDRKITHPTLNDFYLQSHYTHQGMARP
SHYTILYDDIKLTTDEFQCLSNALCYNFQRTTSSVSIPSPTYYAHLICARARMYLNDD
MKLPKVHPNLEKYPMYFM*

>Rhiir2_1|1704186
MQIARVTKNMPAIYYQQFRGPDSVKQAYKDAEKRLNKPPQMILFIIPGEKSPMNTDI
YETIKRVMDTEIGCLSQCISVDSKRNFRNPQYCANLALKINLKLGGVNSSLPEEKLSL
PDGGKVLFLGADVTHPKDKYGSSICAVVGSLDDQAARYVTKYQDQERSGKEEILKI
DKMIKEILKDYCEYQRKKRDLPKDEKDEKVLPPYIIMYRDGVSESQFERVLRFELPKI
KEACAQFRKGNKPYEPKIIFAVVGKRHHTRIFPINPQSKNEADRNGNCLVGTVIDKKI
THPTLNDFYLQSHYANQGTARPSHYTILYDDIKLTIDQFQSLSNTLCYNFQRATSSV
SIPSPTYYAHLTCARAKMYLVSSRVKFVKKDKNDKPPPPKLLKLHRNLNKFPMYFM*

>Rhiir2_1|1450356
MDVNICVLHSDYKVLKIDNRSEECCVDLIVRHTLTDENFVREGAQQEYLVNKRPSL
GTQGRKIHVYTNFFEITSLPKANIYHYDLTITPDVPFLLTRKVFQIFEDSHSKKLKNTP
LVFDGHKNIFSCKPLPLKDSATFDITLPEVDSRSILTKREPRTLKIRLKKIDEINMDELQ
RYLVGKAKRTPNVLNAITFVNALIKHQTLVNYVPSGRSFYTRNGRINLTGIAEAWQG
YYQSARPTPGKMMINVDLTATAFCESGPLINIVVKLLEKRSTNDLRGGINERERNKL
EKELKNYIIRVIHRKTNQFYRILKFTSLSAQQTKFYDADGDIIDVASYFQKSHKCLDYP
YLPCVIAGEGIFLPMEVCEVIENQRYFRKLNDRQTAEIIKLTYQSPHSRAAKIMRGVE
EIDYASEKMTQFNMIVSNKMATVKARILPTPTINYRYSSIVPEYGVWDLKDKKLATG
AILTSWSILVFGTDNEHSVKRFVRELIDACSIAGMNIPNKSPPILHANAQGNVEENLK
KAWLRAGHAANSKPQLIICVLPNTSSQLYGTIKYVGDTIVGVVTQCIQSRYLLEASN
QYYSNVCLKINAKLGGVNSFLTLRQNPFLNEKASILMGADVTHPGVSDDYYSVAAL
CASVDSGASRYAASIRMQHMPRIEIISDLTNMVKDALKVFYQTCGRKPERILFYRDG
VSEYQFKQVLEEEIRAIKSACHSLEQEYSPTITFVIVQKRHHARFFPIDRIDSDRTGN
CLPGTLIETEVVHPVEFDFYLQSQAGLQGTCRPTHYHVLYDENNFTSDSLQTLSYN

LCYTYARCTRAVSIVPSVYYAHLACKRGRFHLRDDNFKESSSSSSTGKITLSTVEPK
LQNVMYFI*

>Rhiir2_1|1478504
MANITEFVKRPGLGRLGRPIRIRTNYFEITNLPASNIHHYDIDISPEVPPTLNRKIFQIA
ENSFYGIRAVFDGRRNVYTIRPLDVHTLDVTLPEDNTIRRPPRIFRIRIKKVNEIHMEE
INRFLNGRGSISSNILTGIMALNILIRHVPSTTLNSVGRIFYTNQDSRPLSGGVEALQG
YFQSIRPTPKKLMINVDLHATAFYESDSLVQLVVKILNKRSADDLRRIHDRDRTKLEK
CLKNLKIYATHDENALNRRFRIFKVTNTSASNTIFDDNGNQTDVASYFQRKYNIQLLY
PFFPCIVIRSGTYLPMEVCNVVEGQRYMRKLNERQTANMSKLTRPPQSHASKINQG
IQILNYQQNQYMQQFDFRVSNEMAITQARILPAPKLQYHPTSRESIFTPRNGLWNLR
DKKVATGATLGSWACAVFGNERDYPMSAIQIFIRELVTTCQDTGMNIPNKNPPIQHC
NPQGPIETSLRQVWVKAGNLAKSKPQLILCILPNTGVPLYAEIKRVSDTVIGVASQCV
QGKHMFAAKKLYCANVCLKMNVKLGGMNSFIDPIQVPFITQRPTILMGASVTHHAP
GAENSGRPSIAAVTASMDAKASRYAASIRVQTGRQEVISDLAEMVKELLKTFYMTC
GRKPDRILFYRDGVSESQFSIVLKDEIKAIKEACKSLDEKYKPTITFVVIQKRHHTRFF
PINARDGDRIGNCPSGTVVETTIVHPFEFDFYLLSHPSLQGTSRPTHYHVLLDENGF
NADSLQTLTYNLCYVFARCTRAVSIVPPVYYAHLVCARARFHASGENWSDPDTSE
GAGGVARYAAVKAELLKVMYFM*

>Rhiir2_1|1478501
MMINIDFHATAFYESSSLTRIVKKVLNKRTIEELRDISERDRLKIENFLKNLKIYATHDE
NALNRRFRISKVTNTSDSNTTTFDDNGNQTDVASYFQRKYNMQLQHPFLPCIVIRE
ETYLPLEVCNVVEQVWLKAGNDAKAQPQLILCILPNTGVSLYDAEIKRVGDTVTGVV
TLCIQSRNMFAIKKQYCVNVYLKINAKLGGMNSFINPSQLLFVSESPTIILGASVIHPV
PGDTSRPSIAAVAASIDAMASRYVASIRVQQEVISHLADMTK*

>Rhiir2_1|1067111
MEELDFQLVKRPGIGDEGKRIRVRANFFEVTRMQREDFSHYDVTTIPKVPQRLNRK
VFDRFVEQNRYGVLRDARPVFDGRANMFAHKPLRFESDSFDVELEEEGTPVSRTR
PPQSFRIRIKKTRDIVVRDLFQFLNARGDMTNNCLMAITGMDIIISHKISAKFPTVRRS
FYNPQVKKSLGGGLEAYQGYYQSARPTMSGRMMINVDLSATAFYESGPLIDIVVKL
LKNIRTLNELRRGLSTNDRHKVEDFIRGLKIKDNHRTGTGRKFKVEGLTPTPASHTT
FDRGDGSEIDVKTYFQNAYNIRLSYPSLPCVVVRGNVYLPMEVCDIVPEQRYMRKL
NKEQTDVMMNFTRQNPSNRANKIQDGLKLLNYRENEHLQQFGMSISDEMAVVNAR
ILPTPTIEYRQSTRQFRVEPKNGVWNLRDKTLAMGATLGSWSVLAFLDEDTLPKPVI
EKFLVEFTNTCHNTGMNIITKKPPIMHANPQGNIEQSLKLAWINAGNAAKSKPQLILC
ILPSKGQELYAEIKRVSDTIIGVPTQCIQREYLKDPKVQYCANVCLKINVKLGGMNSS
LLSKHIKFVDDEPTILIGADVSHPAPGDKESPSYAALCGSIDSKASRYAATIRVQTRR
YEIIVDLGNMVKELLKVFYQSCGHKPRRIIFYRDGVSEGQFNHVLKNELNAIRSACQ
ALGADYKPTITFIVVQKRHHTRFFPMEKQNSDFKSGNCLPGTVVEEGITHPFEFDFY
LLSHPGLLGTSRPTHYHVLYDKNGFDANSLQTLSYNLCYLYVRCTRAVSLVPPVYY
AHLVTNRAKLHSYKESNGEITSKVVKEDLKKVMYFA*

>Rhiir2_1|1849191
MSLLWKLRFCLHFTLQTRGILDWDTQLGVPTQCILLKRLERKNGIDQICSNIFLKVNA
KLGGQNVILTNDQIDFVSSELTMILGADVFHSGRGDNRPSIVAVCASLNSKSY*

>Rhiir2_1|61334

MDSSIAEFVRRPDLGRVGHSIKVRTNYFEITDLPFPKIYHYDIVIIPEVSPTSNRRIIQE
AENSFSGVKAVFDGRRNVYAVRPFPFGDAHNLEVTMPENDMNNVGIMLPRVYKVII
RKVAEIDMREINRFLNGDCSISSNILTGIMALNVLIHHKPSKEHIKVGRIFYTNQGSQL
LDGGMEVWQGYFQSIRPKPRKMMINIDLHATAFYERGSLIQLVVRILGKSTVEDLW
RIQERDRTKLEKCLKNLKIFVVLRFSRHRFRISKLTNTSASSTTFEVNGQQIDIATYFF
STYGRRLQYPFLPCVVVRNETYLPIEICNVVEGQRYMRKLNERQMADMIEFTCQPP
QIRTNTINQGIEILNYRQNEYIYQFGFQISNDMVITQARVLPTPTLYYHPASKEDTFIP
KDGLWNLKNKKVATGATLGSWACAVFGSERDYPIGTIQNFIRELVNTCQVAGMNIP
NRNPPIQHCNPQGGIENSLKQVWVRAGNSAKSNPQLILCILPNTGAPLYAEIKRVCD
TVIGVATQCIQGKHMFSAKKQYCANVCLKINVKLGGMNSFIDPSQMPFITQRPTIIM
GASVIHPAPGEQNTGRSSIAAVTASVDAKAFRYFAAIRIQHGKQVVIDDLAEIIKELLK
AFYQTCGRKPERILFYRDGISENQFLIVRENVIKAINAACKSLDEKYKPTITFVVVQKR
HHTRFFPIDTRRDGDRTGNCPSGTVVDTTVVHPFEFDFYLLSHPSLQGISRPAHYH
VLYDENGFNADSLQTLTYNLCYNFARCTRAVSIVPPVYYARLVCRRARFHVSGEN
WSNRDTLEGIGSASYAVVKSELLNFMYFA*

>Rhiir2_1|1462304
MKRPGFGNKGVPIKIQTNFFEVTNKIPKMKITHYDVTIFPEVPPRLNINRKVFNHFSE
ENQVDLGGVKPVFDGIKNMFTHKPLPFKDRSFYVELEEDNMPMGSKRPFRRFTIRI
RKTRDFFMEDLFRFLHAKGKMTWNCKMAITAMGVIISHEIFTNHPTIRNLFYTSQGAI
PLFGGIEAWPGYYQLVRPTMGKMMINIDSSATTFYEGGPLIQMIVKILGLRSPNDLR
KGLSDSKDRRKVEKIIKNLRISDNHIPENRRNFKIEKLTRSSASDTTINRNKIDVETYF
QKKYKKRLLYPFLPCVVVGKNYYLPIEVCDVIPGQQHIRKLNEIQTADMIKFTCQPPS
ARANKILASLNILDYQNNEYLKQFGMEISNDMTVVNARILPTPIIQYHQTSKENRIEPK
GGEWNLRDKRVATGATLGSWSVLAFLNEKELPDNIIIAFLRKFFITCQDTGMNILNRF
PPICRENGNTEESLRKAWLRAGNNAKVKPQLILCILPHRSVLYGEIKRVSDTVIGVAT
QCVLSKKAKTLKKQYLANICLKINVKLGGENSFLIPEHIQFVADEPTILMGADITHPSL
GGYISPSYAALCGSMNARASRYVASLRVQTGHTEIIKDLKDMVKELLKTFYQTHRLK
PKKILFYRNGVSKSRYIDVLGSELTAIKGACKSLETNYEPTITFVLVKKRRHTRFFPM
ESKYTDRTGNCLPGTVVETDITHPFEFDFYLLSHAGITGTSRPTHYQVLYDENGFDA
DGLQTLSYNLCHTYARCTRAVSLVPPVYYAHLAATRAKFYSSYDFDTGETTFEAVK
EALRKVN*

>Rhiir2_1|1454135
IIEDLKGVVIDLLCVFYQRNQVLPRKFLFYRDGVGETQFQHVKTYEVKALKEVFASV
YRNSGPTLTFIILQKRHHTRFMPTEPRDGDKLGNCSLIFVRMRNLLF*

>Rhiir2_1|1812928
LQVSLEADGPRKAKSFHITIRKIEEINIERLLNYLKGSAQLTGEIQTCVTILNTVLNYKP
RNNFAVVKRGVFPETNDRPKYLYGGIELKTGFCQSMRPAIKLVLGYMIVNVDTCAA
VFHPDGSLLDYVAKILGKRNVEELRRGISDSERRNLERNLKGLIIKVIHRGEKQTKYK
IERLTSQAADFTTFTNKKEGVEMSVADYFAQTYNHSLEFKSLPCLVVKKNLFIPMEV
CDILPGQKYEKTIGDKAKADMIKFTACKPQERFDKIENCIQNIFKHNEDENLRDFEIKI
DPKMMTVDGRVLKSPTVMFNANSKQQNIFPVQNGRWNPKDLVLLRTKPLHNWSV
LVLADRRSCPMEQVKRCMIGLKQKLIEFGMEVGNDPYINYENPQGNIESSLQISVQK
AHNNKSFPPQLLIVIIPRKPSPLYGEVKMIADTKIGIVTQCILAEKLRNPNNKSLWANI
GLKINAKLGGHNCNLIKEELSTIGKVPTMIVGADISHPGVGQKDQPSIAAVCASVEPN
ATTYYGRISVNKQIRNETIECLGEMISDLLKAFYEKNKVLPKRMIFYRDGVSEGQFRS
VLNREVIALKNAFDKIYQKDPPNLTFIIAQKRHHTRFIPINRRDADKLGNCIPGTVVDQ

SIVHCNEFDFYLQSHSGLQGTTRPTHYYVLHDENKFGADEIQNLTYRLCYLYARCS
SAISVVPPIAYAHLLAARARLYPTIEVPGEQNCEESESISSIPLVSKELAKVMYFV*
